# siRNA Delivery via Cross‐Linked Gelatin Microparticles Enables Targeted Modulation of Osteogenic‐Vascular Cross‐Talk: An Advanced Human 3D in Vitro Test System for Therapeutic siRNA

**DOI:** 10.1002/adhm.202504773

**Published:** 2026-01-22

**Authors:** Franziska Mitrach, Jonas Kubat, Stefan Simm, Alexandra H. Springwald, Burak Demir, Anton Liebezeit, Michael C. Hacker, Michaela Schulz‐Siegmund

**Affiliations:** ^1^ Pharmaceutical Technology Medical Faculty Leipzig University Leipzig Germany; ^2^ Institute for Bioanalysis Department of Applied Sciences Coburg University of Applied Sciences and Arts Coburg Germany; ^3^ Institute of Pharmaceutics and Biopharmaceutics Faculty of Mathematics and Natural Sciences Heinrich Heine University Düsseldorf Düsseldorf Germany

**Keywords:** bone regeneration, siRNA release, tissue engineering, spheroids, microspheres

## Abstract

Small interfering RNAs (siRNAs) have drawn particular attention for their ability to transiently and sequence‐specifically silence target genes, not only for systemic but also for localized application. For bone regeneration, targeting inhibitory regulators by siRNAs offers opportunities to improve osteogenic–angiogenic coupling.

Conventional experimental models often oversimplify this interaction as they fail to capture these multicellular tissue dynamics. To address this, we established a human three‐dimensional co‐culture model composed of osteogenic and vascular microtissues embedded in fibrin hydrogels to investigate siRNA effects on microtissue interaction.

Local siRNA delivery to microtissues was achieved by oligomer‐stabilized calcium phosphate nanoparticles (CaP‐NP) loaded onto cross‐linked gelatin microparticles (cGM). siRNA/CaP‐NP‐loaded cGM were assembled with human mesenchymal stem cells (hMSCs) to microtissues. This approach was demonstrated by silencing two antagonists with distinct expression profiles: Chordin, a low‐abundance BMP inhibitor, and WWP‐1, a highly expressed E3 ligase. Only Chordin siRNA improved the osteogenic‐vascular cross‐talk, whereas WWP‐1 siRNA effects were limited to osteogenic effects. Next‐generation sequencing (NGS) supported these results.

We demonstrate that this co‐culture platform permits systematic investigation of siRNA‐mediated modulation of osteogenic–endothelial interactions, offering a relevant human model for preselecting therapeutic siRNA targets to advance vascularized bone tissue regeneration.

## Graphical abstract

Osteogenic‐angiogenic cross‐talk is a vital prerequisite for vascularized bone regeneration. In this study, we investigated the effects of siRNA‐mediated silencing of two inhibitory proteins, Chordin and WWP‐1, via CaP‐NP‐loaded gelatin microparticles in osteogenically differentiated microtissues. NGS data showed prolonged angiogenic signaling in Chordin‐silenced microtissues. A microtissue‐based test system was set up to investigate siRNA for effects on osteogenic‐angiogenic cross‐talk.

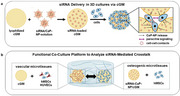

**Abbreviations**
α‐SMA: alpha smooth muscle actin
ALP: alkaline phosphatase
ANGPT2: angiopoietin‐2
Asc: ascorbic acid
β‐Gly: β‐glycerophosphate
BGLAP: bone gamma‐carboxyglutamate protein
BMP‐2: bone morphogenetic protein 2
BMP‐4: bone morphogenetic protein 4
BMPR1B: bone morphogenetic protein receptor type 1B
CaP‐NP: oligomer‐stabilized calcium phosphate nanoparticles
CD31: cluster of differentiation 31
cGM: cross‐linked gelatin microparticles
COL10A1: collagen type X alpha 1 chain
Ct: threshold cycle
CTNNB1: β‐catenin
CXCL5: C‐X‐C motif chemokine ligand 5
CXCL6: C‐X‐C motif chemokine ligand 6
DEED: *N,N*‐diethyl ethylenediamine
DEGs: differentially expressed genes
DMP1: dentin matrix acidic phosphoprotein 1
DNA: deoxyribonucleic acid
EDN1: endothelin‐1
GLI3: GLI family zinc finger 3
GSK3B: glycogen synthase kinase 3 beta
HIF1A: hypoxia inducible factor 1 subunit alpha
hMSCs: human mesenchymal stem cells
HUVECs: human umbilical vein endothelial cells
IBSP: integrin binding sialoprotein
IGF1: insulin‐like growth factor 1
JunB: proto‐oncogene, AP‐1 transcription factor subunit
LRP5: low‐density lipoprotein receptor‐related protein 5
MAPK: mitogen‐activated protein kinase
MAP2K2: mitogen‐activated protein kinase kinase 2
mRNA: messenger RNA
NGS: next‐generation sequencing
NRP2: neuropilin 2
oMT: osteogenic microtissues
oMT^Chrd^
: osteogenic microtissues transfected with Chordin siRNA
oMT^ctrl^
: osteogenic microtissues transfected with control siRNA
oPNMA: oligo(pentaerythritol diacrylate‐co‐N‐isopropylacrylamide‐co‐maleic anhydride)
PECAM‐1: platelet and endothelial cell adhesion molecule 1
pNP: para‐nitrophenol
PTCH1: patched‐1
RANKL: receptor activator of NF‐κB ligand
Runx2: runt‐related transcription factor 2
SD: standard deviation
SERPIN1: serpin family E member 1
siRNA: small interfering RNA
SMAD1: mothers against decapentaplegic homolog 1
SMAD2: mothers against decapentaplegic homolog 2
SMAD9: mothers against decapentaplegic homolog 9
SMURF1: SMAD specific E3 ubiquitin protein ligase 1
TGF‐β: transforming growth factor β
TNFSF11: tumor necrosis factor superfamily member 11
VEGF: vascular endothelial growth factor
vMT: vascular microtissues
WNT5B: Wnt family member 5B
WNT10B: Wnt family member 10B
WNT11: Wnt family member 11
WWP‐1: WW domain containing E3 ubiquitin protein ligase 1

## 1 Introduction

**FIGURE 1:**
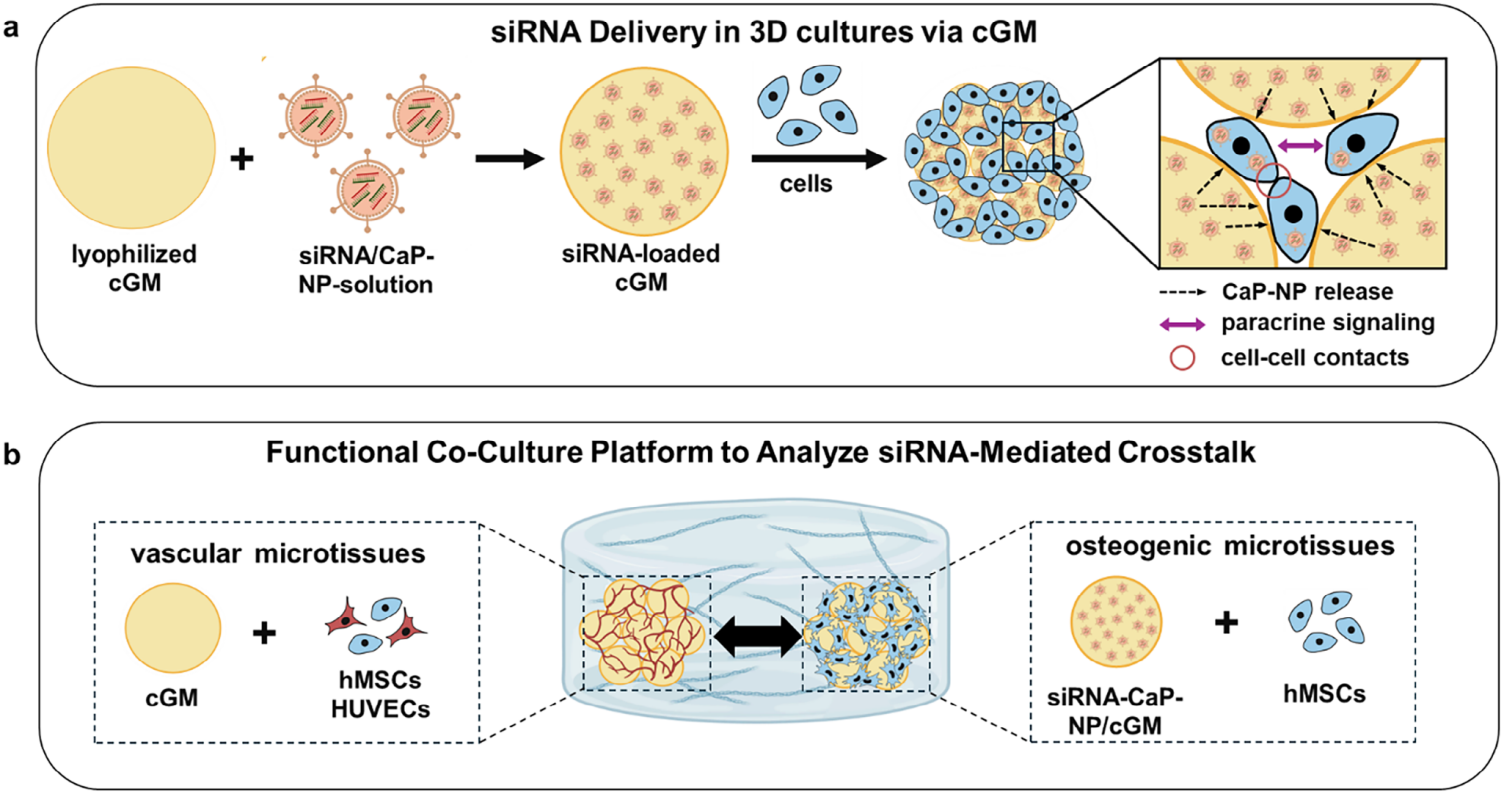
siRNA delivery in 3D cell cultures using cross‐linked gelatin microparticles (cGM) and the associated co‐culture platform for analyzing siRNA‐mediated crosstalk. a) Lyophilized cGM are combined with a solution containing siRNA‐loaded, oligomer‐stabilized calcium phosphate nanoparticles to generate siRNA‐loaded cGM. These siRNA‐loaded microparticles are then assembled with cells into 3D cell cultures, where cells interact with the microparticle platform that releases siRNA locally within the construct. b) A modular co‐culture system of osteogenic and vascular microtissues was designed to explore intercellular communication under siRNA‐mediated modulation. Created in BioRender. Mitrach, F. (2025) *CaP‐NP*: oligomer‐stabilized calcium phosphate nanoparticles; *cGM*: cross‐linked gelatin microparticles; *hMSCs*: human mesenchymal stem cells; *HUVECs*: human umbilical vein endothelial cells; *siRNA*: small interfering RNA.

Nucleic‐acid‐based therapies represent a rapidly advancing field within regenerative medicine, offering the ability to modulate gene expression to direct cellular processes toward therapeutic outcomes [1, 2]. Small interfering RNAs (siRNAs) have gained particular attention due to their sequence‐specific gene silencing capacity [3, 4]. For regenerative applications, local siRNA delivery appears particularly advantageous as it enables transient and targeted gene silencing, promising to modify directly and indirectly the cellular milieu while minimizing systemic exposure [5, 6] (Figure 1).

Among the many areas of application, regeneration of large bone defects stands out as a field where siRNA‐based strategies could address one of the most persistent barriers to clinical success: the necessary vascularization of the regenerating bone tissue [7, 8, 9].

In previous work, we demonstrated that local siRNA can effectively stimulate osteogenic differentiation and formation of bone‐like microtissues [10, 11]. To address large, clinically more relevant bone defects, however, vascularization must be considered, as endothelial cells form networks that supply regenerating tissue [12, 13]. Beyond nutrient supply, there is a cross‐talk between osteogenesis and angiogenesis that includes different kinds of interactions, such as the secretion of growth factors and cytokines by bone‐forming cells as well as angiogenic factors by endothelial cells, crucial for proper bone tissue regeneration [14, 15]. Additionally, cross‐talk also relies on other factors, including extracellular vesicles signaling as well as direct interaction, e.g. via gap junctions [15, 16, 17, 18]. Targeting inhibitory factors of cross‐talk via siRNA thus offers attractive ways to refine these interaction, thereby promoting a more coordinated and effective interplay between the two processes [19, 20]. Consequently, evaluating siRNA effects in bone regeneration requires in vitro co‐culture systems that enable analysis of osteogenic‐endothelial communication.

Angiogenic effects are still predominantly assessed through simple standard in vitro assays, highlighting endothelial cell behavior but neglecting capturing multicellular tissue interactions [14, 21, 22, 23, 24, 25]. Such assays typically include endothelial cell proliferation, migration, and tube formation assays, with the latter often employing human umbilical vein endothelial cells cultured on matrices like Matrigel to mimic capillary‐like structures [14, 21, 22, 23, 24, 25]. Although these assays are rapid, quantitative, and useful for initial screening, they do not capture the complexity of cellular interactions and the long‐term remodeling processes essential for vascularized bone tissue regeneration [14, 21, 22, 23, 24, 25]. They also commonly lack supporting cell types such as pericytes, which are vital for vessel stabilization, maturation, and sustained functionality [14, 21, 22, 23, 24, 25]. Consequently, there is a need for more advanced in vitro models that better replicate these cellular interactions and preserve structural and functional integrity over extended culture periods.

Addressing this demand, 3D microtissue platforms have emerged as a more physiologically relevant alternative, offering the ability to recapitulate native tissue organization, extracellular matrix composition, and cell‐cell signaling [26, 27]. Building on this advantage, efforts have focused on co‐culture systems that seek to combine both osteogenic and vascular development within a single platform [28, 29, 30, 31, 32, 33, 34, 35]. Most approaches rely on relatively complex platforms employing a “hybrid” culture environment that consists of a mixture of osteogenic and vasculogenic media [29, 30, 31, 36]. However, such mixed conditions compromise tissue maturation, as osteogenic media hinder vessel formation while vasculogenic media impair osteogenic differentiation [29, 30, 31]. To address this, an alternative strategy has emerged: microtissues are separately precultured to induce appropriate lineage commitment before being combined into multimodular constructs [32]. This modular approach allows for optimized differentiation of each cell type prior to assembly, thereby reducing the conflicting effects often encountered in conventional co‐culture systems and facilitating the investigation of osteogenic‐angiogenic interactions [32].

Although multimodular approaches represent a significant advancement in co‐culture design, delivering siRNA effectively within 3D constructs remains challenging. siRNA–nanoparticle complexes often exhibit limited tissue penetration beyond the outer layers due to biological and physical barriers—including extracellular matrix density, tight cell–cell junctions, and 3D cellular layering [37, 38, 39, 40, 41]. Additionally, necrotic core formation within central regions of spheroids/microtissues further impairs tissue viability and siRNA delivery efficiency [42, 43, 44]. Thus, how can we optimize siRNA delivery strategies in 3D tissue constructs to enhance silencing efficiency while tackling the issues of limited cell viability? Addressing this challenge requires delivery systems that not only sustain cell viability but also enable efficient siRNA transfection within 3D environments.

Cell‐adhesive cross‐linked gelatin microparticles (cGM) provide such a platform by supporting tissue viability and simultaneously serving as localized siRNA carriers to achieve uniform transfection within 3D constructs [10, 11].

During microtissue formation, cells attach to multiple siRNA‐loaded cGM, forming close cell–cell and cell–material interactions. This architecture minimizes diffusion distances, enabling efficient and homogeneous siRNA transfection. Unlike bulk hydrogels, cGM create discrete, extracellular matrix‐like niches that enhance cell interactions, distribution, and matrix bridging [45, 46, 47]. Structural heterogeneity in microtissues, consisting of cGM and attached cells, supports localized diffusion and concentration gradients of siRNA and signaling molecules, better mimicking the dynamic bone microenvironment than homogeneous bulk hydrogels. Consequently, cGM improves local bioavailability of siRNA and cell‐material interactions crucial for establishing the bone‐vascular interface [45, 46, 47].

In previous work, cGM loaded with siRNA/Lipofectamine RNAiMax complexes achieved a gradual silencing of the BMP‐2 antagonist Chordin in osteogenic microtissues, thereby promoting osteogenic differentiation and reducing necrosis in human mesenchymal stem cell‐derived microtissues [10, 11]. Building on our previous siRNA‐cGM single‐culture systems that enabled lineage‐specific osteogenic control, this study expands the focus to the cross‐talk with endothelial microtissues. We established a 3D human co‐culture model that captures the dynamic interplay between osteogenic and endothelial cells during siRNA‐mediated modulation within a microenvironment. This model overcomes the limitations of 2D, Matrigel‐based, and single‐lineage systems by enabling mechanistic analysis of bidirectional signaling underlying bone‐vascular coupling.

A second task this study focused on is how to overcome the inherently limited loading capacity of cGM. Lipid‐based transfection reagents such as Lipofectamine are limited by the maximal concentrations, preventing aggregation as well as potential toxicity of high Lipofectamine concentrations. The resulting limited loading of cGM with siRNA complexes can restrict dosing precision and transfection efficiency. Therefore, employing delivery systems with higher siRNA loading capacity would be advantageous to achieve more effective and sustained gene silencing in complex multicellular models. Among the multitude of transfection systems, exosome‐based systems serve as essential mediators of cell–cell communication, especially for bone regeneration, and hold great potential as endogenous siRNA carriers [48, 49, 50]. Nevertheless, their large‐scale production, heterogeneous composition, and limited siRNA loading efficiency remain major challenges for reproducible therapeutic applications [48, 49, 50]. In a previous study, however, we found that oligomer‐stabilized calcium phosphate nanoparticles (CaP‐NP) can serve as highly efficient, biocompatible siRNA carriers for reliable gene silencing [51]. A significant improvement of CaP‐NP is their production via a simple precipitation step in the presence of a stabilizing oligomer and their ability to be up‐concentrated via ultrafiltration to achieve the necessary high siRNA concentration for cGM loading without compromising transfection efficiency. Employing CaP‐NP was revealed as a scalable and reliable siRNA delivery platform and a prerequisite for silencing higher expressed targets in this study.

The modular cGM design further permits independent pre‐differentiation of osteogenic and endothelial microtissues before co‐culturing, preventing lineage interference. The co‐culturing in a spatially defined fibrin hydrogel enabled mimicking the spatiotemporal coordination of vascularized bone formation. Together, these conceptual, technological, and structural innovations establish a mechanistically advanced, human‐relevant platform for investigating therapeutic siRNA effects in bone regeneration. In our study, we included two antagonists with distinct expression levels and functions: Chordin, a lower expressed BMP antagonist that blocks activation of the BMP‐2/Smad pathway, and WWP‐1, a higher expressed E3 ubiquitin ligase that promotes proteasomal degradation of key osteogenic transcription factors such as Runx2 and JunB, thereby suppressing downstream differentiation. In order to determine downstream effects of silencing Chordin or WWP‐1, we investigated transcriptomic changes indicating modified osteogenic and angiogenic signaling by next‐generation sequencing.

Finally, we hypothesize that integrating osteogenic and vascular microtissues within a hydrogel matrix will establish a self‐sustaining microenvironment in which both osteogenic and endothelial functions are preserved, and vascular networks remain stable beyond the transient nature of conventional angiogenesis assays. This platform will allow systematic screening of siRNAs modulating osteogenic–vascular cross‐talk and, in the long term, may enable the generation of autologous microtissues pre‐stimulated via siRNA‐loaded cGM. The siRNA targets can be tailored to address individual patient needs, paving the way for personalized microtissues that may serve as modular building blocks for bone regeneration therapies.

## 2 Materials and Methods

### 2.1 Fabrication of Cross‐Linked Gelatin Microparticles

Gelatin microparticles cross‐linked with *N,N*‐diethyl ethylenediamine (DEED)‐derivatized oligo (pentaerythritol diacrylate monostearate‐*co*‐*N*‐isopropylacrylamide‐*co*‐maleic anhydride) (oPNMA) (cGM) were fabricated according to the previously described protocol [11]. In brief, 5 g gelatin (from bovine skin, type B, 225 Bloom, Sigma Aldrich, Seelze, Germany) was dissolved in 45 mL water at 60  °C. Then, gelatin solution was added dropwise to 200 mL Kollisolv MCT 70 (Caesar & Loretz GmbH, Hilden, Germany) at 60  °C, and the resulting mixture was emulsified at 800 rpm for 10 min. The W/O emulsion was cooled on ice and stirred for 30 min, followed by the addition of 100 mL cold acetone and stirring for an additional 1 h. The resulting pristine gelatin microparticles (GM) were washed several times with fresh acetone and dried. Subsequently, GM were cross‐linked with DEED‐derivatized oligomers, including oPNMA‐5, oPNMA‐7.5 and oPNMA‐10 at a GM‐to‐cross‐linker ratio of 1:2 (corresponding equivalent to 10% w/v oPNMA‐x) to achieve varying crosslinking densities. Prior to cross‐linking, oligomer derivatization was achieved by reacting a required amount of DEED (corresponding to 25% theoretical derivatization of anhydride units, mol/mol) with 2 g oPNMA‐x in 18 mL acetone for 2 h. For the cross‐linking step, this solution was then added to the GM that were dispersed in 2 mL acetone. Subsequently, 1 mL of triethylamine (TEA) and 10 mL of demineralized water were introduced, and the reaction mixture was stirred at 800 rpm for 4 h. cGM were washed with acetone and dried under the fume hood overnight. Afterwards, cGM were vacuum‐dried for one day.

### 2.2 Formation and Cultivation of Osteogenic Microtissues

#### 2.2.1 Cell Culture

Human mesenchymal stem cells (hMSCs, Lonza, Basel, Switzerland) were cultivated in DMEM low glucose (Sigma‐Aldrich, Seelze, Germany) with 10% (v/v) fetal bovine serum (Sigma‐Aldrich, Seelze, Germany), 1% (v/v) MEM non‐essential amino acid Solution (Sigma‐Aldrich, Seelze, Germany), and 1% (v/v) penicillin/streptomycin (Sigma‐Aldrich, Seelze, Germany) with a density of 5.000 cells cm^−2^. Cells were cultivated at 37 °C in a humidified cell culture incubator (Binder GmbH, Tuttlingen, Germany) with 5% carbon dioxide. Medium was changed three times a week. Subcultivation was done until 90% confluency was achieved using trypsin/EDTA. Cell passages 3–5 were used for experiments.

#### 2.2.2 Preparation of siRNA‐Loaded Cross‐Linked Gelatin Microparticles

Cross‐linked gelatin microparticles were loaded with siRNA via oligomer‐stabilized calcium phosphate nanoparticles. These were prepared with a total volume of 1,875 µL via the co‐precipitation method according to a previously described protocol based using the stabilizing oligomer o14PEGMA(1:1:2.5)_NH_3_ [51]. In brief, 625 µL of a phosphate solution containing 140 mmol L^−1^ NaCl (AppliChem GmbH, Darmstadt, Germany), 3.75 mmol/L Na_2_HPO_4_ (AppliChem GmbH, Darmstadt, Germany), and 50 mmol L^−1^ HEPES (AppliChem GmbH, Darmstadt, Germany) pH 7.0 was first mixed with 625 µL 40 µmol L^−1^ o14PEGMA(1:1:2.5)_NH_3_ in 50 mmol L^−1^ HEPES pH 7.0 (AppliChem GmbH, Darmstadt, Germany). This mixture was added to 625 µL calcium solution containing 25 µg siRNA (Dharmacon, Lafayette, CO, USA) (2.5 mol L^−1^ CaCl_2_ (AppliChem GmbH, Darmstadt, Germany): 62.5 µL; 20 µmol L^−1^ siRNA: 93.75 µL; 10 mmol L^−1^ Tris pH 7.0 (AppliChem GmbH, Darmstadt, Germany): 468.75 µL). Both solutions were gently mixed and incubated for a minimum of 30 min at room temperature to produce the final siRNA‐loaded CaP‐NP. Afterwards, CaP‐NP were transferred to Amicon Ultra‐4 100K centrifugal filter units (Merck Millipore, Tullagreen, Ireland) and centrifuged (Centrifuge 5430 R, Eppendorf, Hamburg, Germany) at 2000 rcf for 6 min to concentrate the nanoparticles to a volume of 50 µL. For loading of cGM, concentrated nanoparticles were added to lyophilized cGM and vortexed (ROTILABO Mini Vortex, Carl Roth GmbH & Co. KG, Karlsruhe, Germany) until cGM was uniformly covered and appeared transparent. Before further use, loaded cGM were incubated at room temperature for 45 min. Subsequently, loaded cGM were resuspended DMEM low glucose (Sigma‐Aldrich, Seelze, Germany) supplemented with 10% (v/v) fetal bovine serum (Sigma‐Aldrich, Seelze, Germany) and 1% (v/v) MEM non‐essential amino acids (Sigma‐Aldrich, Seelze, Germany) without antibiotics. Table 1 gives an overview of the loading amount and final concentrations of siRNA and cGM per 100 µL cell culture medium.

**TABLE 1:** Final amounts of cGM and siRNA per 100 µL.

Loading amounts		Cell culture medium for resuspension	Final amount/100 µL		
*cGM*	*Concentrated CaP‐NP*		*siRNA*	*cGM*	*CaP‐NP*
3.2 mg	50 µL	5000 µL	0.5 µg	0.064 mg	2 µL
	100 µL		1 µg		4 µL
3.2 mg	50 µL	2500 µL	0.5 µg	0.128 mg	2 µL
	100 µL		1 µg		4 µL

As controls, cGM were loaded with DMEM low glucose (Sigma‐Aldrich, Seelze, Germany) only or AllStars Negative Control siRNA (control siRNA, Qiagen, Hilden, Germany) to exclude off‐target effects (Table 2).

**TABLE 2:** Sense and antisense sequences of Chordin and WWP‐1 siRNA.

siRNA	Sequence (5′‐3′)
siChordin	Sense G.G.U.G.C.A.C.A.U.A.G.C.C.A.A.C.C.A.A Antisense U.U.G.G.U.U.G.G.C.U.A.U.G.U.G.C.A.C.C.
siWWP‐1	Sense G.G.A.G.G.C.G.C.U.U.A.U.A.U.G.U.A.A.U.U.U Antisense A.U.U.A.C.A.U.A.U.A.A.G.C.G.C.C.U.C.C.U.U

#### 2.2.3 Assembly of cGM and hMSCs to Osteogenic Microtissues

For assembly with the cGM, hMSCs were detached with trypsin/EDTA (Sigma‐Aldrich, Seelze, Germany) and cell numbers were determined via trypan blue (Sigma‐Aldrich, Seelze, Germany). 100 µL of single cell suspension (10^5^ cells mL^−1^) were added to 100 µL cGM suspension in DMEM low glucose (Sigma‐Aldrich, Seelze, Germany) supplemented with 10% (v/v) fetal bovine serum (Sigma‐Aldrich, Seelze, Germany), 1% (v/v) MEM non‐essential amino acid solution (Sigma‐Aldrich, Seelze, Germany) without antibiotics in one well of a BIOFLOAT 96‐well U‐bottom spheroid plate (Sarstedt, Nümbrecht, Germany). To ensure a fast and uniform seeding of hMSCs and cGM, an E3 multipette (Eppendorf, Hamburg, Germany) was used. After seeding, plates were gently shaken on an orbital shaker (Heidolph Unimax 1010, Heidolph Instruments GmbH & Co. KG, Schwabach, Germany) for 1 min at 110 rpm. Cells were cultivated at 37 °C in a humidified cell culture incubator (Binder GmbH, Tuttlingen, Germany) with 5% carbon dioxide. After 24 h, the medium was changed to 100 µL osteogenic medium based on DMEM low glucose (Sigma‐Aldrich, Seelze, Germany) supplemented with 10% (v/v) fetal bovine serum (Sigma‐Aldrich, Seelze, Germany), 1% (v/v) MEM non‐essential amino acid solution (Sigma‐Aldrich, Seelze, Germany), 1% (v/v) penicillin/streptomycin (Sigma‐Aldrich, Seelze, Germany), 100 ng mL^−1^ dexamethasone (Sigma‐Aldrich, Seelze, Germany), 50 µg mL^−1^ ascorbic acid (Sigma‐Aldrich, Seelze, Germany) and 10 mmol L^−1^ β‐glycerophosphate disodium salt hydrate (Sigma‐Aldrich, Seelze, Germany). Since Chordin is a bone morphogenetic protein 2 (BMP‐2) antagonist, 100 ng mL^−1^ recombinant human BMP‐2 (R&D Systems, Wiesbaden, Germany) were added to osteogenic medium for Chordin siRNA experiments. Medium was changed three times a week by complete removal of culture medium and replaced by 100 µL freshly prepared osteogenic medium.

### 2.3 Formation and Cultivation of Vascular Microtissues

#### 2.3.1 Cell Culture

Human umbilical vein endothelial cells from pooled donors (HUVECs, PromoCell, Heidelberg, Germany) were cultivated in endothelial cell growth medium (PromoCell, Heidelberg, Germany) with a density of 10 000 cells cm^−2^ according to the manufacturer's protocol. Cells were cultivated at 37 °C in a humidified cell culture incubator (Binder GmbH, Tuttlingen, Germany) with 5% carbon dioxide. Cell culture medium was changed three times a week. Subcultivation was done until > 70% confluency using DetachKit (PromoCell, Heidelberg, Germany) according to the manufacturer's protocol. Cell passages 3 – 6 were used for experiments.

Human mesenchymal stem cells (hMSCs, Lonza, Basel, Switzerland) were cultivated in DMEM low glucose (Sigma‐Aldrich, Seelze, Germany) with 10% (v/v) fetal bovine serum (Sigma‐Aldrich, Seelze, Germany), 1% (v/v) MEM non‐essential amino acid Solution (Sigma‐Aldrich, Seelze, Germany), and 1% (v/v) penicillin/streptomycin (Sigma‐Aldrich, Seelze, Germany) with a density of 5000 cells cm^−2^. Cells were cultivated at 37 °C in a humidified cell culture incubator (Binder GmbH, Tuttlingen, Germany) with 5% carbon dioxide. Medium was changed three times a week. Subcultivation was done until 90% confluency was achieved using trypsin/EDTA. Cell passages 3–5 were used for experiments.

#### 2.3.2 Assembly of cGM and hMSCs to Vascular Microtissues

To fabricate vascular microtissues, cGM were suspended in endothelial cell growth medium (PromoCell, Heidelberg, Germany) with a density of 1.28 mg mL^−1^ and 100 µL of this cGM suspension was added per well of a BIOFLOAT 96‐well U‐bottom spheroid plate (Sarstedt, Nümbrecht, Germany) to obtain a cGM amount of 0.128 mg per well. hMSCs (Lonza, Basel, Switzerland) and HUVECs (PromoCell, Heidelberg, Germany) were detached using trypsin/EDTA, and the respective cell numbers were determined using trypan blue (Sigma‐Aldrich Seelze, Germany). With a cell density of 10^5^ cells mL^−1^ hMSC and HUVECs were mixed in different ratios in endothelial cell growth medium (PromoCell, Heidelberg, Germany) and 100 µL of mixed cell suspension (10 000 cells) was added per well to the cGM. To ensure a fast and uniform seeding of hMSCs and cGM, E3 multipette (Eppendorf, Hamburg, Germany) was used. After seeding, plates were gently shaken on an orbital shaker (Heidolph Unimax 1010, Heidolph Instruments GmbH & Co. KG, Schwabach, Germany) for 1 min at 110 rpm. Cells were cultivated at 37 °C in a humidified cell culture incubator (Binder GmbH, Tuttlingen, Germany) with 5% carbon dioxide. After 24 h, the medium was removed and exchanged with 100 µL endothelial cell growth medium 2 (PromoCell, Heidelberg, Germany) to induce vascular differentiation. Medium was changed three times a week by complete removal of culture medium and replacing by 100 µL endothelial cell growth medium 2 (PromoCell, Heidelberg, Germany).

### 2.4 Gene Expression Analysis

Total RNA from single microtissues was isolated with RNAqueous‐micro total RNA isolation kit (Invitrogen, Darmstadt, Germany) including DNA digestion according to the manufacturer's instructions. Quantities and purities of RNA samples were determined using the Synergy H1 plate reader (BioTek, Bad Friedrichshall, Germany) and Take3 microvolume plate (BioTek, Bad Friedrichshall, Germany). About 2 µL RNA were measured per sample and 2 µL of elution solution served as reagent blank. Until analysis, RNA was stored at – 20 °C. Gene expression levels were determined by using 1‐Step quantitative real‐time PCR using TaqMan gene expression assay (Applied Biosystems, Thermo Fisher Scientific, Waltham, MA, USA) or PrimePCR SYBR green assay (Bio‐Rad Laboratories Inc., Feldkirchen, Germany). For TaqMan gene expression assay, 45 ng total RNA (total volume: 35 µL) was mixed with 2.5 µL TaqMan gene expression assays (Applied Biosystems, Thermo Fisher Scientific, Waltham, MA, USA) and 12.5 µL TaqMan Fast Virus 1‐Step master mix for qPCR (Applied Biosystems, Thermo Fisher Scientific, Waltham, MA, USA) with a total volume of 50 µL in Multiplate PCR plates (Bio‐Rad Laboratories Inc., Feldkirchen Germany). Used TaqMan gene expression assays are summarized in Table 3. For PrimePCR SYBR green assay, iTaq Universal SYBR green 1‐Step kit (Bio‐Rad Laboratories Inc., Feldkirchen, Germany) was used according to the manufacturer's instructions. Briefly, 45 ng total RNA (total volume: 8.75 µL) was mixed with 11.25 µL Reaction mix (10 µL 20x iTaq Universal SYBR green 1‐Step Kit, 0.25 µL iScript reverse transcriptase, 1 µL 20x PrimePCR SYBR green assay) in a total volume of 20 µL Multiplate PCR plates (Bio‐Rad Laboratories Inc., Feldkirchen Germany). The used Prime PCR SYBR green assays are summarized in Table 3. Quantitative real‐time PCR was performed with CFX96 Touch real‐time PCR detection system (Bio‐Rad Laboratories Inc., Feldkirchen, Germany). 60s acidic ribosomal protein P0 (RPLP0) was used as a housekeeping gene since it is very stable during osteogenic differentiation of human mesenchymal stem cells. For gene expression analysis of vascular microtissues, glyceraldehyde‐3‐phosphate dehydrogenase (GAPDH) was used as housekeeping gene. A no‐template control (NTC, negative control well that contains water instead of a sample) was included to exclude genomic DNA contamination of reagents. Gene expression levels were calculated using the ΔΔC_t_ method. The efficiency of the respective TaqMan gene expression assay was analyzed using a 4‐fold serial dilution and was included in calculations.

**TABLE 3:** Gene expression assays used in this study.

Assay	Symbol	Gene Name	Assay ID
TaqMan^TM^ gene expression assay	*α‐SMA*	alpha smooth muscle actin	Hs00426835_g1
	*CHRD*	Chordin	Hs00415315_m1
	*GAPDH*	glyceraldehyde‐3‐phosphate dehydrogenase	hs02786624_g1
	*IBSP*	integrin‐binding sialoprotein	Hs00173720_m1
	*RPLP0*	60S acidic ribosomal protein P0	Hs99999902_m1
	*VEGFA*	vascular endothelial growth factor	Hs00900055_m1
PrimePCR SYBR green assay	*WWP‐1*	ubiquitin E3 ligase	qHsaCED0043974
	*RPLP0*	60S acidic ribosomal protein P0	qHsaCED0038653

The quantitative real‐time PCR conditions are summarized in Table 4.

**TABLE 4:** Quantitative real‐time PCR conditions.

Assay	Step	Temperature	Time	Cycles
TaqMan^TM^ gene expression assay	reverse transcription	50 °C	5 min	1
	reverse transcription inactivation/initial denaturation	95 °C	20 s	1
	denature	95 °C	15 s	40
	annealing	60 °C	60 s	40
PrimePCR SYBR Green Assay	reverse transcription	50 °C	10 min	1
	reverse transcription inactivation/initial denaturation	95 °C	1 min	1
	denature	95 °C	10 s	40
	annealing	60 °C	30 s	40
	melt‐curve analysis	65–95 °C 0.5 °C increment 5 s/step		

### 2.5 Cell Viability

#### 2.5.1 DNA Content

Cell culture medium was removed, and samples were washed with warm PBS (37 °C). Afterwards, microtissues were transferred to a 0.5 mL reaction tube (Sarstedt, Nümbrecht, Germany) containing 100 µL lysis buffer consisting of Tris‐EDTA buffer with 50 µg mL^−1^ proteinase K and 0.02% sodium dodecyl sulfate (SDS). Samples were incubated for 5 h at 56 °C using Eppendorf ThermoMixer C (Eppendorf, Hamburg, Germany). DNA content of lysed samples was quantified using Quant‐iT PicoGreen dsDNA assay (Invitrogen, Darmstadt, Germany) according to the manufacturer's protocol. Briefly, 20 µL of sample and 80 µL of Tris‐EDTA buffer were mixed in a black 96‐well plate (Corning, NY, USA). 100 µL of the aqueous working solution of Quant‐iT PicoGreen dsDNA reagent (1:200 dilution in Tris‐EDTA buffer) was added to the samples and incubated in the dark for 5 min at room temperature on an orbital shaker (Heidolph Unimax 1010, Heidolph Instruments GmbH & Co. KG, Schwabach, Germany). As reagent blank, 100 µL Tris‐EDTA buffer without sample was incubated with 100 µL of the aqueous working solution of Quant‐iT PicoGreen dsDNA reagent. Fluorescence intensities of samples were measured using Synergy H1 plate reader (BioTek, Bad Friedrichshall, Germany) at 480/520 nm. Fluorescence value of reagent blank was subtracted from that of each sample and DNA amounts of the samples were calculated using a DNA standard curve (Invitrogen, Darmstadt, Germany) in a range of 0.001 – 10 µg mL^−1^.

#### 2.5.2 WST‐8 Assay

Cell viability was determined using Rotitest Vital (Carl Roth, Karlsruhe, Germany) according to the manufacturer's protocol. Briefly, 10 µL of undiluted Rotitest Vital reagent was added per 100 µL cell culture medium, and cells were incubated with reagent for 4 h at 37 °C and 5% CO_2_. Afterwards, supernatants were collected and transferred to a black 96‐well plate (Corning, NY, USA). Formazan absorbance was measured at 450 nm using Synergy H1 plate reader (BioTek, Bad Friedrichshall, Germany). As reagent blank, 100 µL cell culture media without cells were incubated with 10 µL undiluted Rotitest Vital reagent. The viability (%) was calculated by using formula:

(1) $$\mathrm{Cell}\;\mathrm{viability}\;\left(\%\right)=\frac{\left(\mathrm{Absorbance}\;\mathrm{of}\;\mathrm{sample}-\mathrm{Absorbance}\;\mathrm{of}\;\mathrm{reagent}\;\mathrm{blank}\right)}{\left(\mathrm{Absorbance}\;\mathrm{of}\;\mathrm{control}\;\mathrm{group}-\mathrm{Absorbance}\;\mathrm{of}\;\mathrm{reagent}\;\mathrm{blank}\right)}\times 100\%$$

### 2.6 Alkaline Phosphatase Activity Quantification

Alkaline phosphatase activity as an early marker of osteogenic differentiation was quantified using p‐nitrophenyl phosphate assay which relies on the dephosphorylation of colorless p‐nitrophenyl phosphate to yellow p‐nitrophenyl. Cell culture medium was removed, and samples were washed with 100 µL warm PBS (37 °C). For cell lysis, microtissues were transferred to a 0.5 mL reaction tube (Sarstedt, Nümbrecht, Germany) containing 100 µL 0.1% triton X‐100 (AppliChem GmbH, Darmstadt, Germany) and incubated for 45 min on ice. For measurements, samples were diluted in a ratio of 1:2 with 0.1% triton X‐100 (AppliChem GmbH, Darmstadt, Germany) and kept on ice. 10 µL of the respective diluted sample was transferred to a transparent 96‐well plate (Corning, NY, USA). Immediately after, 100 µL alkaline phosphatase yellow (pNPP) liquid substrate system for ELISA (Sigma‐Aldrich, Seelze, Germany) was added to 10 µL sample. To avoid evaporation of samples during measurement, the plate was covered with a transparent plate sealer (Corning, NY, USA). Alkaline phosphatase activity was determined by quantification of p‐nitrophenyl absorbance every 5 min over a period of 2 h at 37 °C and 405 nm using Synergy H1 plate reader (BioTek, Bad Friedrichshall, Germany). As a reagent blank, 10 µL 0.1% Triton X‐100 (AppliChem GmbH, Darmstadt, Germany) was incubated with 100 µL alkaline phosphatase yellow (pNPP) liquid substrate system for ELISA (Sigma‐Aldrich, Seelze, Germany). Before the calculation of enzyme activity, absorbance values of reagent blank were subtracted from those of each sample, and enzyme activity was calculated using a p‐nitrophenyl (Honeywell International Inc., NC, USA) standard curve in a range of 6.25 – 400 µmol L^−1^. The alkaline phosphatase activity was normalized to the DNA content.

### 2.7 Calcium Quantification

Mineralization of microtissues as a later marker of osteogenic differentiation was determined via calcium quantification using the Arsenazo III method, which forms blue complexes with calcium ions. For this, cell culture medium was removed and the microtissues were washed with 100 µL warm PBS (37 °C). For cell lysis, microtissues were transferred to a 0.5 mL reaction tube (Sarstedt, Nümbrecht, Germany) containing 0.5 mol L^−1^ HCl (AppliChem GmbH, Darmstadt, Germany). For mechanical lysis, microtissues were incubated at 1000 rpm for 8 h at room temperature using Eppendorf ThermoMixer C (Eppendorf, Hamburg, Germany) following an overnight incubation at 250 rpm on an orbital shaker (Heidolph Unimax 1010, Heidolph Instruments GmbH & Co. KG, Schwabach, Germany) at room temperature. The next day, samples were incubated for 2 h in an ultrasonication bath (Transonic 460 H, Elma Schmidbauer GmbH, Singen, Germany) followed by a further 8 h incubation at 2000 rpm and room temperature using Eppendorf ThermoMixer C (Eppendorf, Hamburg, Germany) and an overnight shaking at 250 rpm using orbital shaker (Heidolph Unimax 1010, Heidolph Instruments GmbH & Co. KG, Schwabach, Germany). The next day, samples were finally measured. For this, a 10 µL sample was mixed with 990 µL Calcium AS FS ready‐to‐use reagent (DiaSys Diagnostic Systems GmbH, Holzheim, Germany) in a 1.5 mL reaction tube (Sarstedt, Nümbrecht, Germany), vortexed for 2 s, and incubated for 5 min at room temperature. Afterwards, samples were transferred to semi‐microcuvettes (Sarstedt, Nümbrecht, Germany) and the absorbance of calcium‐arsenazo III complexes was measured using Genesys 6 UV/Vis spectrophotometer (Thermo Electron Corp., MA, USA) at 650 nm. As reagent blank, 10 µL 0.5 mol L^−1^ HCl (AppliChem GmbH, Darmstadt, Germany) was incubated with 990 µL calcium AS FS ready to use reagent (DiaSys Diagnostic Systems GmbH, Holzheim, Germany) and absorbance value was subtracted from sample values. To calculate calcium concentrations, a calcium standard curve (DiaSys Diagnostic Systems GmbH, Holzheim, Germany) in a range of 6.25 – 100 µg mL^−1^ was prepared.

### 2.8 VEGF Secretion

To analyze secretion of VEGF by osteogenic microtissues, cell culture supernatants were collected on days 3, 5, 7, 9, 12, 14, 16, 19, 21, 23, 26, and 28 of osteogenic differentiation and stored at −20 °C. The amount of VEGF secreted was quantified by using a solid phase sandwich ELISA Kit (Human VEGF DuoSet ELISA, R&D Systems, Wiesbaden, Germany) according to the manufacturer's protocol. Cell culture medium without cells served as blank. Absorbance values were detected at 450 nm using Synergy H1 plate reader (BioTek, Bad Friedrichshall, Germany). To correct optical imperfections in the plate, samples were measured at 570 nm and the values were subtracted from the 450 nm values. Secreted VEGF amounts/day were calculated using a standard curve in a range from 2000 – 31.3 pg mL^−1^ recombinant human VEGF standard (R&D Systems, Wiesbaden, Germany).

### 2.9 Illumina Next‐Generation Sequencing

Total RNA from single microtissues was isolated with RNAqueous‐micro total RNA isolation kit (Invitrogen, Darmstadt, Germany), including DNA digestion according to the manufacturer's instruction. Illumina Next‐Generation Sequencing was performed by the Core Unit DNA technologies of Leipzig University. In order to find differentially expressed mRNA genes (DEGs) the reads were mapped on the genome of homo sapiens (ENSEMBL, GRCh38.p14), and feature counts were performed on the assigned gtf. We then performed differential expression analysis for each RNA type for every tissue individually by first normalizing and then comparing mutant vs. wildtype counts using DESeq2 [52]. Genes were considered “differentially expressed” if the adjusted p‐value was lower than 0.05. Clustering of genes and the following visualization was done using the ComplexHeatmap package in R [53] using filtered sets of genes for osteogenic and angiogenic. Principal Component Analysis was used for dimensionality reduction on mRNAs, either with all samples or individually for each condition, using scikit‐learn in Python [54]. Visualization was done using the Python package plotnine [55].

### 2.10 Co‐Culture of Osteogenic and Vascular Microtissues

#### 2.10.1 Combination of Microtissues in Fibrin Hydrogel

To analyze interactions, osteogenic and vascular microtissues were prepared and pre‐differentiated as described above for 14 days. On day 15, both microtissues were combined using a fibrin hydrogel. Fibrin hydrogels were prepared by combining equal amounts of 2,21% (w/v) NaCl (AppliChem GmbH, Darmstadt, Germany) in 1x PBS, 20 mmol L^−1^ CaCl_2_ (AppliChem GmbH, Darmstadt, Germany) in dH_2_O, 250 KIU mL^−1^ aprotinin (Sigma‐Aldrich, Seelze, Germany) in 1x PBS, 40 mg mL^−1^ fibrinogen from human plasma with ≥80% of clottable protein (Sigma‐Aldrich, Seelze, Germany) in 1x PBS and 2.5 U mL^−1^ thrombin from human plasma (Sigma‐Aldrich, Seelze, Germany) in 1x PBS on ice to avoid early gelation of gel. Hydrogel solution was then vortexed (Rotilabo mini vortex, Carl Roth GmbH + Co. KG, Karlsruhe, Germany), and 50 µL of solution were added per well of a BIOFLOAT 96‐well U‐bottom spheroid plate (Sarstedt, Nümbrecht, Germany) using E3 multipette (Eppendorf, Hamburg, Germany). To allow gelation, plates were incubated for 30 min humidified cell culture incubator (Binder GmbH, Tuttlingen, Germany) at 37 °C and 5% carbon dioxide. Subsequently, osteogenic and vascular microtissues were added on the gelled fibrin hydrogels and further 50 µL of hydrogel solution were added via E3 multipette (Eppendorf, Hamburg, Germany) to embed microtissues. To allow gelation, plates were incubated for 15 min humidified cell culture incubator (Binder GmbH, Tuttlingen, Germany) at 37 °C and 5% carbon dioxide. Finally, 150 µL endothelial cell growth medium 2 (PromoCell, Heidelberg, Germany) were added per hydrogel, and co‐cultures were incubated until analysis in a humidified cell culture incubator (Binder GmbH, Tuttlingen, Germany) at 37 °C and 5% carbon dioxide. Medium was changed three times a week by complete removal of culture medium and replacing it with 150 µL endothelial cell growth medium 2 (PromoCell, Heidelberg, Germany).

#### 2.10.2 Analysis of Vascular Outgrowth and Mineralization

Cell culture medium was removed, and fibrin hydrogels were transferred to a 2.0 mL reaction tube (Sarstedt, Nümbrecht, Germany). After washing with warm 1x PBS (37 °C), fibrin hydrogels were then fixed with 10% paraformaldehyde (Thermo Fisher Scientific, Darmstadt, Germany) for 1 h at room temperature. To visualize hydroxyapatite formation, the Osteoimage mineralization assay (Lonza, Basel, Switzerland) was performed according to the manufacturer's instructions. To visualize cell morphology and outgrowth of cells from microtissues to hydrogel, samples were stained afterwards overnight with 2.5 µL 400x Alexa Fluor 568 Phalloidin (Thermo Fisher Scientific, Waltham, MA, USA) per 1 mL 2% bovine serum albumin (Sigma‐Aldrich, Seelze, Germany) in 1x PBS (Sigma‐Aldrich, Seelze, Germany) in the dark. Before imaging, samples were washed twice with 1x PBS (Sigma‐Aldrich, Seelze, Germany) for 5 min each. For imaging confocal microscope Leica TCS SP8 and LAS X software (Leica, Wetzlar, Germany) were used.

##### 2.10.2.1 Image Analysis

Images were analyzed using ImageJ software, version 1.54 (National Institutes of Health, Bethesda, MD, USA) using the Angiogenesis Analyzer and Analyze Skeleton plugins. Fluorescence intensities of Osteoimage staining and the radial outgrowth of vascular structures from microtissues were quantified in ImageJ too. Vascular outgrowth was defined as the distance from the microtissue boundary to the most distal point of the extending vascular structures within the hydrogel.

#### 2.10.3 Immunohistochemistry

##### 2.10.3.1 Cryosectioning

Samples were washed twice with PBS (37 °C) and fixed with 10% paraformaldehyde (Thermo Fisher Scientific, Darmstadt, Germany) for 16 h at 4 C. After removal of paraformaldehyde, samples were washed twice with PBS and dehydrated by using an increasing sucrose gradient consisting of 10%, 20%, 30% and 40% sucrose for 30 min each at room temperature. Afterwards, samples were embedded in tissue freezing medium (Leica, Wetzlar, Germany) for 1 h at ‐80 °C. Until cryosectioning, frozen samples were stored at ‐20 °C. Cryosections of 30 – 50 µm thickness were prepared by using a Leica cryostat CM 1950 (Leica, Wetzlar, Germany). Cryosections were collected on Superfrost Plus gold adhesion microscope slides (Epredia Netherlands B.V., Breda, Netherlands). Before staining, the slides were air‐dried overnight and stored at room temperature in the dark.

##### 2.10.3.2 Staining Procedure

For immunohistochemical staining of the endothelial marker CD31/PECAM‐1, a blood vessel staining kit based on Peroxidase System (Merck Millipore, Tullagreen, Ireland) was used according to the manufacturer's instructions. First, tissues were rehydrated by dipping of slides into the specified staining dishes according the following procedure: 1) 100% ethanol (Carl Roth GmbH + Co. KG, Karlsruhe, Germany) ‐ 2 x 2 min; 2) 95% ethanol ‐ 2 x 2 min; 3) 70% Ethanol – 1 x 2 min; 4) 50% ethanol – 1 x 2 min; 6) 1x IHC select TBS rinse buffer (Part No. 90218). To recover tissue antigenicity, epitope retrieval was done by incubation of slides in sodium citrate buffer pH 6.0 containing 10 mmol/L sodium citrate (Carl Roth GmbH + Co. KG, Karlsruhe, Germany) and 0.05% Tween 20 (AppliChem GmbH, Darmstadt, Germany) for 25 min at 95 °C. Slides were removed and cooled down for 15 min at room temperature. Then, the area around the tissue was encircled by using a PAP pen (DakoCytomation Denmark A/S, Glostrup, Denmark) to create a water‐repellent barrier around the tissues to ensure that aqueous staining reagents remain on the tissue. Afterwards, 100 µL of 3% (v/v) hydrogen peroxide (AppliChem GmbH, Darmstadt, Germany) was added to the sample to block endogenous peroxidase activity. Sections were incubated for 20 min in a hydrated incubation enclosure and rinsed 2 x for 5 min in 1x IHC select TBS rinse buffer (Part No. 90218). Afterward, 3 drops of the ready‐to‐use blocking reagent (Part No. 90219) was added per sample and incubated for 30 min in a hydrated incubation enclosure at room temperature. To remove the Blocking Reagent, slides were gently rinsed for 15 s with 1x IHC select TBS rinse buffer (Part No. 90218) while holding the slides at 45° angle. Immediately after that, 50 µL of mouse anti‐CD31/PECAM‐1 monoclonal antibody (1:200; Part No. 90214) was applied per sample and incubated for 2 h in a hydrated incubation enclosure at room temperature. Slides were rinsed 3x for 5 min in 1x IHC select TBS rinse buffer (Part. No. 90218), and samples were covered with 3 drops of the ready‐to‐use goat anti‐mouse secondary antibody solution (Part No. 21538). Slides were incubated for 15 min at room temperature in a hydrated incubation enclosure. Unbound secondary antibody was removed by rinsing of slides 3x for 5 min in 1x IHC select TBS rinse buffer (Part No. 90218). Then, 3 drops of the ready‐to‐use streptavidin‐HRP solution (Part No. 72002) were added per sample, and slides were incubated for 15 min in a hydrated incubation enclosure at room temperature. The streptavidin‐HRP solution was removed, rinsing of slides 3x for 5 min in 1x IHC select TBS rinse buffer (Part No. 90218). Afterward, DAB chromogen A (Part No. 20778) and DAB chromogen B (Part No. 20779) were mixed in a 1:25 ratio, and 100 µL of this freshly prepared chromogen reagent was added per sample, followed by an incubation of 10 min in a hydrated incubation enclosure at room temperature. Chromogen reagent was removed by rinsing of the section under tap water for 5 min. To coverslip the slides, 1 drop of aqueous mounting medium for IHC (Abcam, Cambridge, United Kingdom) was added to tissue and coverslips (Labsolute, Th. Geyer, Renningen, Germany) were applied. Nikon Eclipse TE2000S‐inverted microscope (Nikon Corporation, Tokyo, Japan) was used for imaging.

### 2.11 Statistics

Experimental data were analyzed using OriginPro 2019 (version 9.6.0.172, OriginLab Corporation, Northampton, MA, USA). Unless otherwise specified, all experiments were conducted with a minimum of four independent replicates (n ≥ 4). Results are presented as mean ± standard deviation). Statistical differences between groups were evaluated using one‐way or two‐way analysis of variance followed by Tukey's post hoc test for multiple comparisons. Analysis was done using a significance level of *p* < 0.05 which was set as a minimal level of statistical significance. Statistically significant differences between groups are indicated by a single asterisk (*) in figures and tables. Each figure legend includes details on sample size (n), statistical test used, data presentation, and the meaning of significance symbols.

## 3 Results

### 3.1 Optimization of siRNA Delivery via Cross‐Linked Gelatin Microparticles (cGM) Using Oligomer‐stabilized Calcium Phosphate Nanoparticles (CaP‐NP)

In the first phase of our study, we focused on optimizing the system by identifying the ideal conditions for loading cGM with siRNA via CaP‐NP. To achieve this, we systematically investigated the influence of key parameters—including the cross‐linking degree of cGM, the amount of cGM used, and the loading volume of CaP‐NP. By carefully varying these factors, we were able to define a set of conditions that enable efficient siRNA delivery within osteogenic microtissues.

However, we first needed to consider the practical challenge of efficiently loading the cGM with sufficient amounts of CaP‐NP, aiming for a total siRNA dose of 1 µg/microtissue, as this dose had previously been proven effective in monolayer transfection [51]. Achieving such a high local siRNA concentration within microtissues required a strategy to increase nanoparticle concentration without affecting their siRNA loading capacity. We finally resolved this issue by applying ultrafiltration, which allowed us to concentrate the siRNA‐loaded CaP‐NP without compromising their siRNA loading.

#### 3.1.1 Influence of Cross‐Linking Degree and CaP‐NP Loading Amount

In order to assess the impact of the cross‐linking degree, Chordin siRNA‐loaded CaP‐NP were incorporated into three cGM formulations with high (10% oPNMA‐12.5^+DEED^), medium (10% oPNMA‐7.5^+DEED^), and low (10% oPNMA‐5^+DEED^) cross‐linking degrees. Microscopical analysis showed the formation of crystal‐like structures upon loading of CaP‐NP to cGM with high cross‐linking degree, indicating that the CaP‐NP became unstable within the highly cross‐linked matrix (Figure S1). In contrast, no such crystal formation was observed in cGM formulation with low and medium cross‐linking degrees (Figure S1). Based on these findings, cGM with a high cross‐linking degree was excluded from further investigations.

**FIGURE 2:**
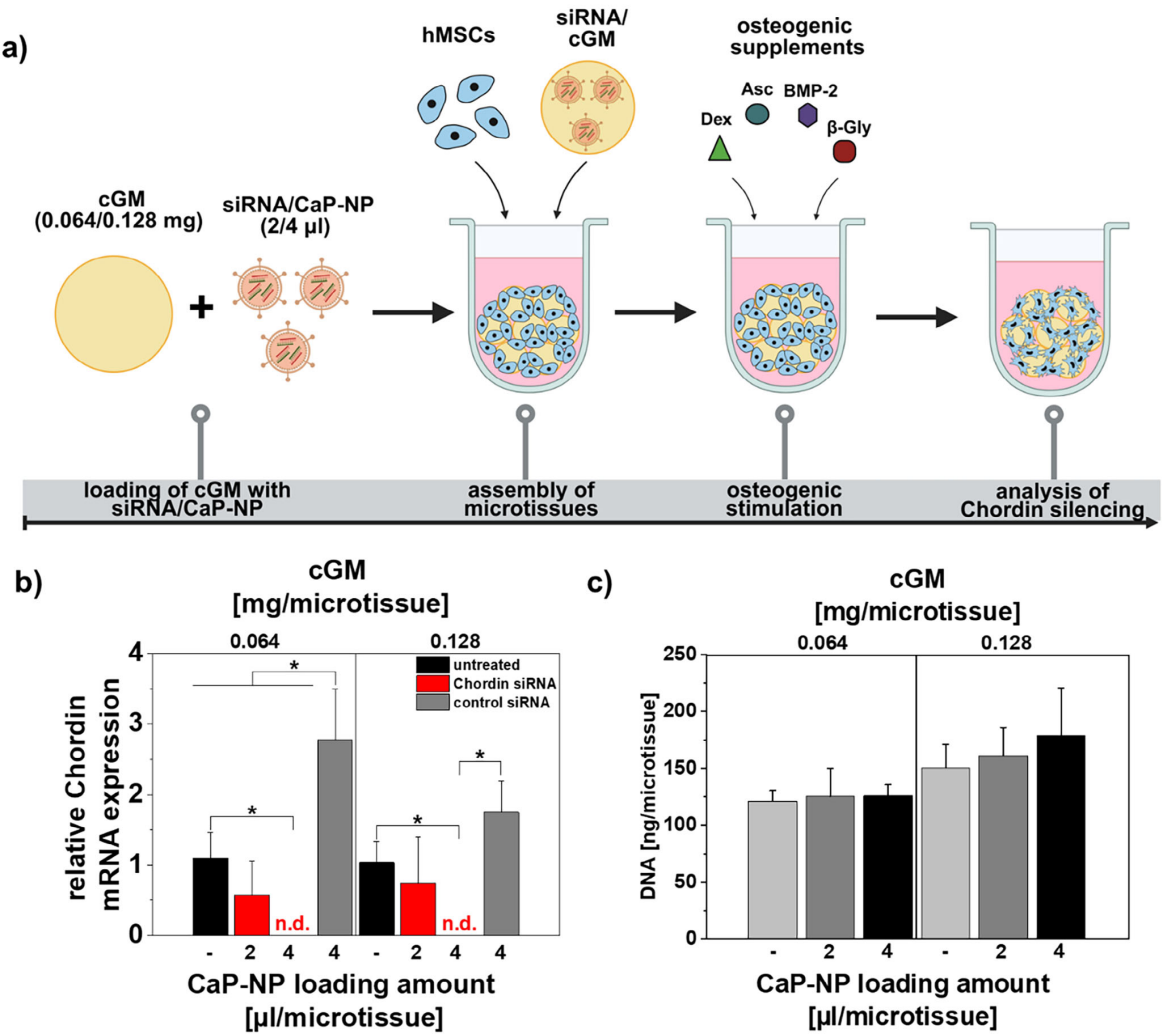
Analysis of siRNA‐mediated Chordin silencing efficiency in osteogenic microtissues upon loading of cGM with a low cross‐linking degree (5%–10%) with siRNA‐carrying CaP‐NP. a) Experimental set up: 1875 µL of siRNA‐loaded CaP‐NP were prepared and concentrated via ultrafiltration to a volume of 50 µL. Afterwards, 1.6 mg cGM (0.064 mg cGM/microtissue) or 3.2 mg cGM (0.128 mg cGM/microtissue) were loaded with 50 (2 µL CaP‐NP/microtissue) or 2 x 50 µL (4 µL CaP‐NP/microtissue) of concentrated CaP‐NP. About 10 000 hMSCs were then aggregated with 0.064 or 0.128 mg cGM, and osteogenic differentiation was induced by addition of osteogenic supplements. Created in BioRender. Mitrach, F. (2025) b) Chordin silencing efficiency was quantified via gene expression levels using one‐step quantitative real‐time PCR at day 4 of osteogenic differentiation and showed a dose‐dependent effect of CaP‐NP loading. Highest Chordin silencing was obtained for loading of cGM with 4 µL concentrated CaP‐NP and was used together with a cGM amount of 0.128 mg/microtissue for further experiments. c) DNA content of microtissues as a measure of cell proliferation was investigated at day 7. Results showed no adverse effects of CaP‐NP loading on cell proliferation. Data are presented as mean ± SD (*n* = 4). Statistically significant differences are indicated with (∗) between the different groups (*p* < 0.05), two‐way ANOVA with Tukey's post hoc test. *Asc*: ascorbic acid; *BMP‐2*: bone morphogenetic protein 2; *β‐Gly*: beta‐glycerophosphate; *CaP‐NP*: oligomer‐stabilized calcium phosphate nanoparticles; *cGM*: cross‐linked gelatine microparticles; *Dex*: dexamethasone; *DNA*: deoxyribonucleic acid; *hMSCs*: human mesenchymal stem cells; *mRNA*: messenger RNA; *n.d*.: not detectable; *siRNA*: small interfering RNA.

In the initial experiments, 0.064 mg of cGM per microtissue—an amount previously shown to be effective—was loaded with concentrated CaP‐NP formulations to achieve the final concentrations. Each microtissue received either 2 µL (0.5 µg siRNA) or 4 µL (1 µg siRNA) of concentrated CaP‐NP suspension. The detailed loading procedure and corresponding final concentrations per microtissue are provided in greater detail in Table 1 of the Materials and Methods section. Interestingly, no Chordin silencing was observed with the medium cross‐linking degree, regardless of the nanoparticle loading volume (Figure S2). In contrast, successful downregulation was achieved using cGM with a low cross‐linking degree, and this effect was highly dependent on the CaP‐NP loading amount. These dose‐dependent effects were confirmed by gene expression analysis of the BMP‐2 antagonist Chordin, demonstrating a clear correlation between the CaP‐NP loading on cGM and silencing efficiency. Loading with 2 µL of siRNA‐carrying CaP‐NP resulted in approximately 50% silencing, with no significant difference between the cGM formulations. However, higher standard deviations indicated that this lower dose did not achieve uniform or efficient siRNA delivery across microtissues. In contrast, loading with 4 µL of siRNA‐loaded CaP‐NP led to a complete downregulation of *Chordin* compared to controls, highlighting the importance of adequate nanoparticle distribution for effective gene silencing (Figure 2b).

#### 3.1.2 Influence of cGM Amount

Having successfully achieved Chordin silencing in osteogenic microtissues using cGM with a low cross‐linking degree, we next sought to determine whether the silencing efficiency depends on the amount of cGM applied per microtissue. Since the higher cGM dose of 0.128 mg per microtissue had not been tested before, we first evaluated its impact on key parameters such as cell proliferation and osteogenic differentiation markers. Previous assessments revealed that increasing the cGM amount promotes enhanced osteogenic differentiation, and no adverse effects on DNA content were observed (Figure 2c, Figure S3). Based on these findings, we included the 0.128 mg cGM dose in the silencing study to investigate whether it could further improve Chordin knockdown efficacy and osteogenic differentiation. For comparison, we maintained the same CaP‐NP loading volumes of 2 µL and 4 µL as used with 0.064 mg cGM per microtissue. Remarkably, similar to the lower cGM dose, complete downregulation of *Chordin* expression was observed when loading 4 µL of concentrated CaP‐NP onto 0.128 mg cGM (Figure 2b). This indicates that, while higher cGM amounts sustain potent gene silencing, they also provide an improved microenvironment that supports osteogenic activity.

Based on this systematic investigation, the optimized conditions were defined as 0.128 mg of cGM with a low cross‐linking degree (10% oPNMA‐5^+DEED^) loaded with 4 µL of concentrated CaP‐NP per microtissue. These parameters were applied for all subsequent analyses.

### 3.2 Effects of siRNA‐Mediated Silencing of Chordin and WWP‐1 on Osteogenic Differentiation of Microtissues

Building on the optimized conditions for siRNA delivery via cGM, we investigated in the next step the biological impact of Chordin siRNA‐mediated mRNA knockdown on osteogenic differentiation of the microtissues. To further evaluate the versatility of our system, we extended our investigation by selecting the ubiquitin E3 ligase WWP‐1 as a novel target that promotes the degradation of key osteogenic transcription factors.

#### 3.2.1 Chordin siRNA‐Mediated Effects on Osteogenic Differentiation

**FIGURE 3:**
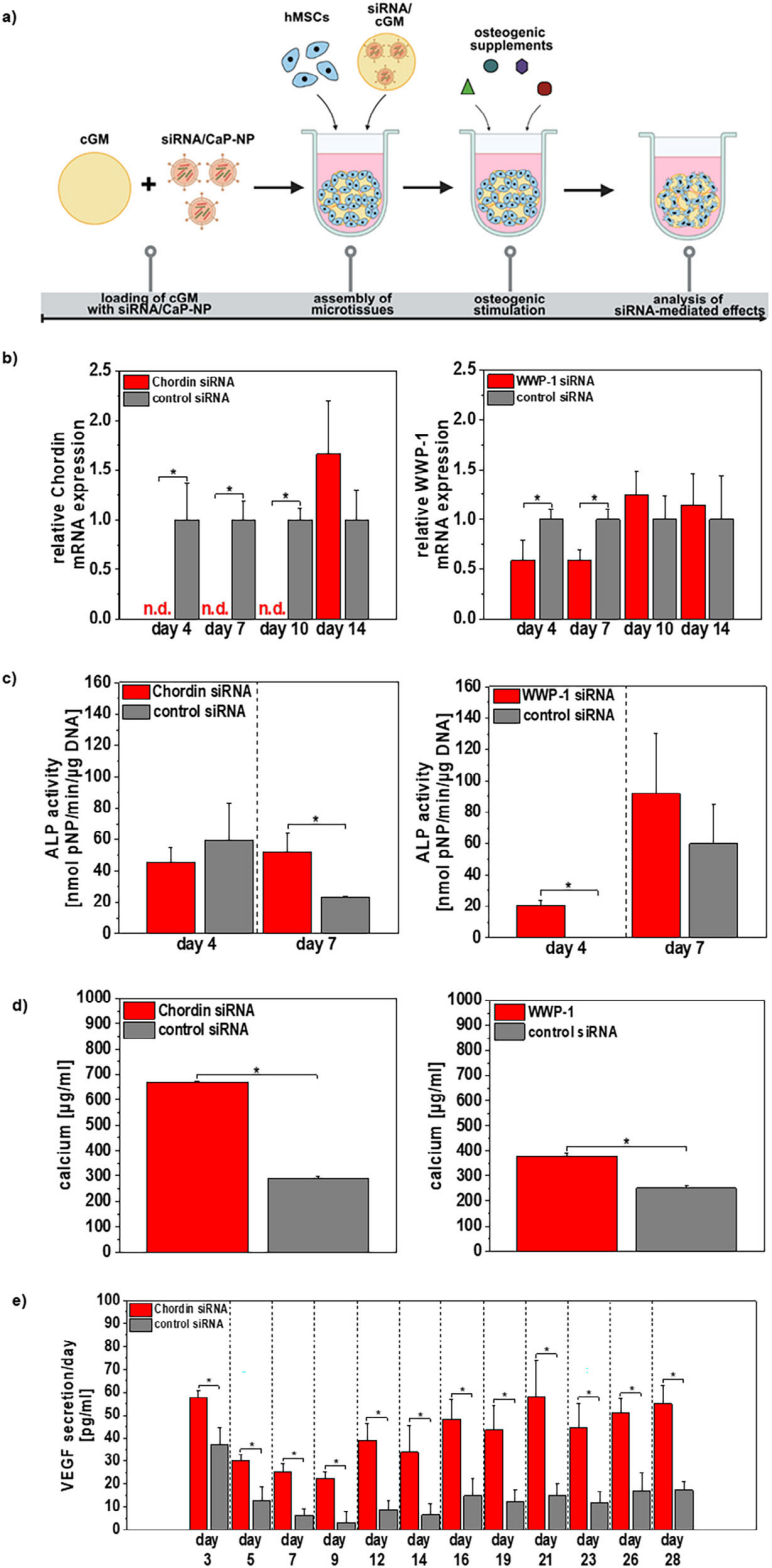
Analysis of siRNA silencing efficiency in osteogenic microtissues upon loading of cGM with siRNA‐carrying CaP‐NP and analysis of effects on osteogenic differentiation. a) Experimental setup: 1875 µL of siRNA‐loaded CaP‐NP were prepared and concentrated via ultrafiltration to a volume of 50 µL. Afterwards, 3.2 mg cGM (0.128 mg cGM/microtissue) were loaded with 100 µL of concentrated CaP‐NP. 10 000 hMSCs were then aggregated with 0.128 mg cGM, and osteogenic differentiation was induced by the addition of osteogenic supplements. Created in BioRender. Mitrach, F. (2025) b) siRNA‐mediated silencing efficiency of Chordin and WWP‐1 was quantified via gene expression levels over a period of 14 days of osteogenic differentiation and showed successful downregulation of both antagonists in osteogenic microtissues. c) Downstream effects of siRNA‐mediated antagonist silencing in microtissues were quantified via alkaline phosphatase activity on days 4 and 7, as well as d) calcium content as a measure of mineralization on day 18. e) Secretion of the important pro‐angiogenic factor VEGF by microtissues was quantified in cell culture supernatants using ELISA over 28 days of osteogenic differentiation. Data are presented as mean ± SD (*n* = 4). Statistically significant differences are indicated with (∗) between the different groups (*p* < 0.05), one‐way ANOVA with Tukey post hoc test. *ALP*: alkaline phosphatase; *CaP‐NP*: oligomer‐stabilized calcium phosphate nanoparticles; *cGM*: cross‐linked gelatin microparticles; *DNA*: deoxyribonucleic acid; *hMSCs*: human mesenchymal stem cells; *mRNA*: messenger RNA; *n.d*.: not detectable; *pNP*: para‐nitrophenyl; *siRNA*: small interfering RNA; *VEGF*: vascular endothelial growth factor.

Silencing efficiency of BMP‐2 antagonist Chordin was analyzed via gene expression analysis and resulted in a complete downregulation of *Chordin* in comparison to control groups (Figure 3b). This high silencing efficiency was maintained over a period of 10 days, leading to a significantly enhanced osteogenic differentiation. On day 7, we found significantly increased activity of the early marker of osteogenic differentiation, alkaline phosphatase, whereas no significant difference was measured on day 4. Quantification of calcium as a measure for mineralization on day 18 showed a strong increase as a consequence of siRNA‐mediated Chordin silencing (Figure 3c,d). We further found that Chordin siRNA led to a significantly increased secretion of VEGF which is an important pro‐angiogenic factor influencing osteogenesis‐angiogenesis interaction (Figure 3e).

In comparison to Lipofectamine RNAiMax, which was used in previous studies of our group for siRNA loading of cGM [10, 11], CaP‐NP showed a higher siRNA loading capacity resulting in stronger silencing efficiency, supporting oligomer‐stabilized CaP‐NP as an advantageous transfection reagent for siRNA in our cGM based microtissue system.

#### 3.2.2 WWP‐1 siRNA‐Mediated Effects on Osteogenic Differentiation

Analysis of the silencing efficiency of WWP‐1 siRNA showed a successful decrease in *WWP‐1* expression in osteogenic microtissues. Osteogenic microtissues treated with WWP‐1 siRNA showed a 40% lower *WWP‐1* mRNA expression in comparison to microtissues treated with control siRNA (Figure 3b).

In comparison to Chordin siRNA treatment, we found that WWP‐1 silencing caused an earlier upregulation of ALP. On day 4, microtissues treated with WWP‐1 siRNA indicated a significantly increased ALP activity in comparison to control group, whereas significant differences in the transiently upregulated enzyme were not observed on day 7 anymore (Figure 3c). Calcium quantification on day 18 further showed significantly increased mineralization of microtissues in response to WWP‐1 siRNA treatment (Figure 3d). Contrary to Chordin siRNA treatment, siRNA‐mediated WWP‐1 silencing led to no further increased VEGF secretion by microtissues; notably, WWP‐1 experiments were performed without BMP‐2, as WWP‐1, in contrast to Chordin, is not a BMP‐2 antagonist.

### 3.3 Effects of siRNA‐Mediated Silencing of Chordin and WWP‐1 on Downstream Signaling Events

Next‐generation sequencing of osteogenic microtissues was performed to analyze a broad range of transcriptional changes. Samples were analyzed at day 7 to characterize the immediate, direct impact of siRNA treatment of Chordin or WWP‐1 knockdown in downstream signaling events. In contrast, transcriptional changes at day 14 were assessed to determine whether the siRNA‐mediated knockdown of both negative regulators translated into a persistent enhancement of osteogenic differentiation and angiogenic stimulators after the silencing was diminished.

#### 3.3.1 Effect of Chordin siRNA

**FIGURE 4:**
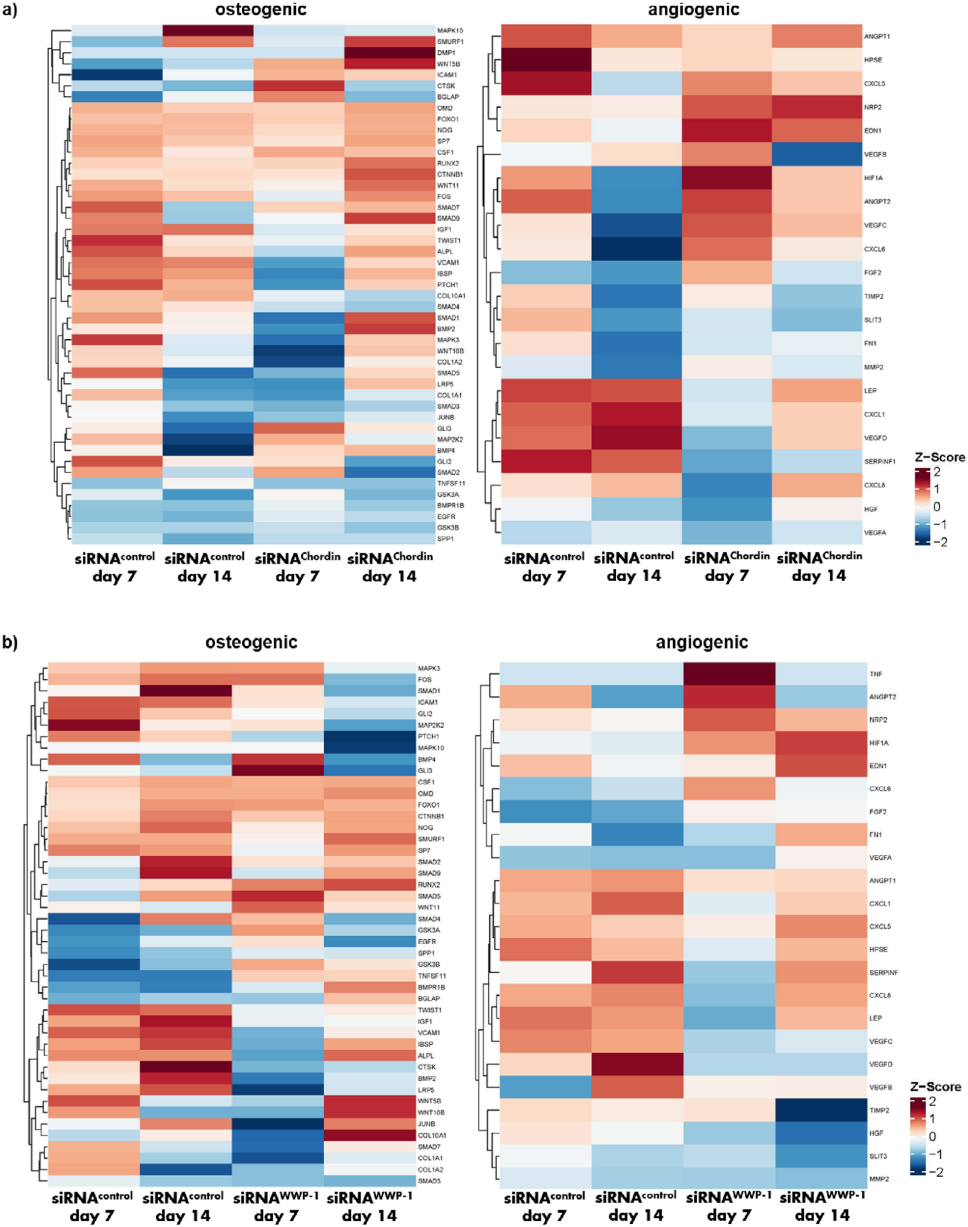
Heat maps illustrating the expression profiles of osteogenic and angiogenic marker genes identified by next‐generation sequencing in osteogenic microtissues at days 7 and 14 in response to a) siRNA‐mediated Chordin silencing and b) siRNA‐mediated WWP‐1 silencing. Differential expression patterns highlight temporal regulation of genes associated with osteogenic differentiation and vascularization processes.

siRNA‐mediated silencing of Chordin led to a broad transcriptional upregulation of osteogenic markers at day 14, including for example *SMURF1*, *SMAD1*, *SMAD9*, *LRP5*, *RUNX2*, *CTNNB1*, and *DMP1*, pointing to a concerted activation of BMP, canonical and non‐canonical Wnt, and p38 MAPK signaling. *WNT5B*, *WNT11*, *BMP4*, *MAP2K2*, and *GLI3* were already increased at day 7 and remained elevated at day 14 over osteogenic microtissues treated with control siRNA, suggesting early induction of non‐canonical WNT, BMP, p38 MAPK, and Hedgehog signaling in response to siRNA‐mediated Chordin silencing, which may lead to an elevated RUNX2‐mediated osteogenic differentiation. Interestingly, *TNFSF11*, also known as RANKL, was reduced at day 14, whereas the osteocyte marker *DMP1* was increased, indicating a potential shift toward bone formation (Figure 4a).

Chordin knockdown in osteogenic microtissues also increased several pro‐angiogenic markers, including *CXCL5*, *CXCL6*, *NRP2*, *EDN1*, *HIF1A*, *ANGPT2*, *VEGFB*, and *VEGFC* at day 7 and/or day 14, pro‐angiogenic markers associated with an influence on chemotactic recruitment and support of angiogenesis of endothelial and lymphatic cells. Meanwhile, *SERPIN1*, an anti‐angiogenic factor, was reduced at both time points, thereby alleviating endogenous suppression of these processes (Figure 4a).

#### 3.3.2 Effect of WWP‐1 siRNA

In contrast, WWP‐1 silencing resulted in a mixed osteogenic profile, with reduced expression of *SMAD1*, *SMAD2*, *SMAD9*, *MAP2K2*, *PTCH1*, *IGF1*, *IBSP*, *BMP2*, and *LRP5*, indicating attenuation of BMP, TGF‐β, Hedgehog, p38 MAPK, IGF1, and canonical Wnt signaling components. At the same time, WWP‐1 knockdown increased *RUNX2*, *SMAD5*, *WNT11*, *WNT5B*, *WNT10B*, *GSK3B*, *TNFSF11*, *BMPR1B*, *BGLAP*, and *COL10A1* at day 7 and/or day 14, suggesting a compensatory shift toward RUNX2‐driven differentiation, non‐canonical and canonical Wnt activity, osteoclast‐regulating signals, and markers associated with matrix mineralization and endochondral ossification (Figure 4b).

For angiogenic markers, WWP‐1 silencing increased *ANGPT2*, *HIF1A*, *EDN1*, and *CXCL5*, indicating preserved or enhanced support for vascular maturation, hypoxia‐responsive VEGF induction, and endothelial recruitment despite the changes in osteogenic pathways. In contrast, *VEGFC* and *VEGFD* were consistently decreased at both time points (Figure 4b).

### 3.4 Development of Vascular Microtissues With cGM as Cell‐Adhesive Material

Following the demonstration that siRNA‐mediated Chordin and WWP‐1 silencing in osteogenic microtissues enhanced differentiation, our next focus was to engineer vascular microtissues enabling us to investigate interactions between osteogenic and vascular cells in response to siRNA treatment, creating a more physiologically relevant bone tissue model.

To this end, we established co‐cultures from hMSCs and HUVECs. HUVECs serve as a reproducible endothelial model closely resembling clinically relevant outgrowth endothelial cells, while hMSCs are expected to function as pericyte‐like cells, supporting and stabilizing vascular structures.

We first optimized this system by assessing the impact of cGM as an adhesive scaffold and by evaluating various hMSC:HUVEC ratios reported in the literature to ensure stable and viable vascular microtissue formation [32, 35, 56]. Through microscopic and gene expression analyses, we identified conditions that favor uniform vascular network development and effective endothelial‐pericyte interactions.

**FIGURE 5:**
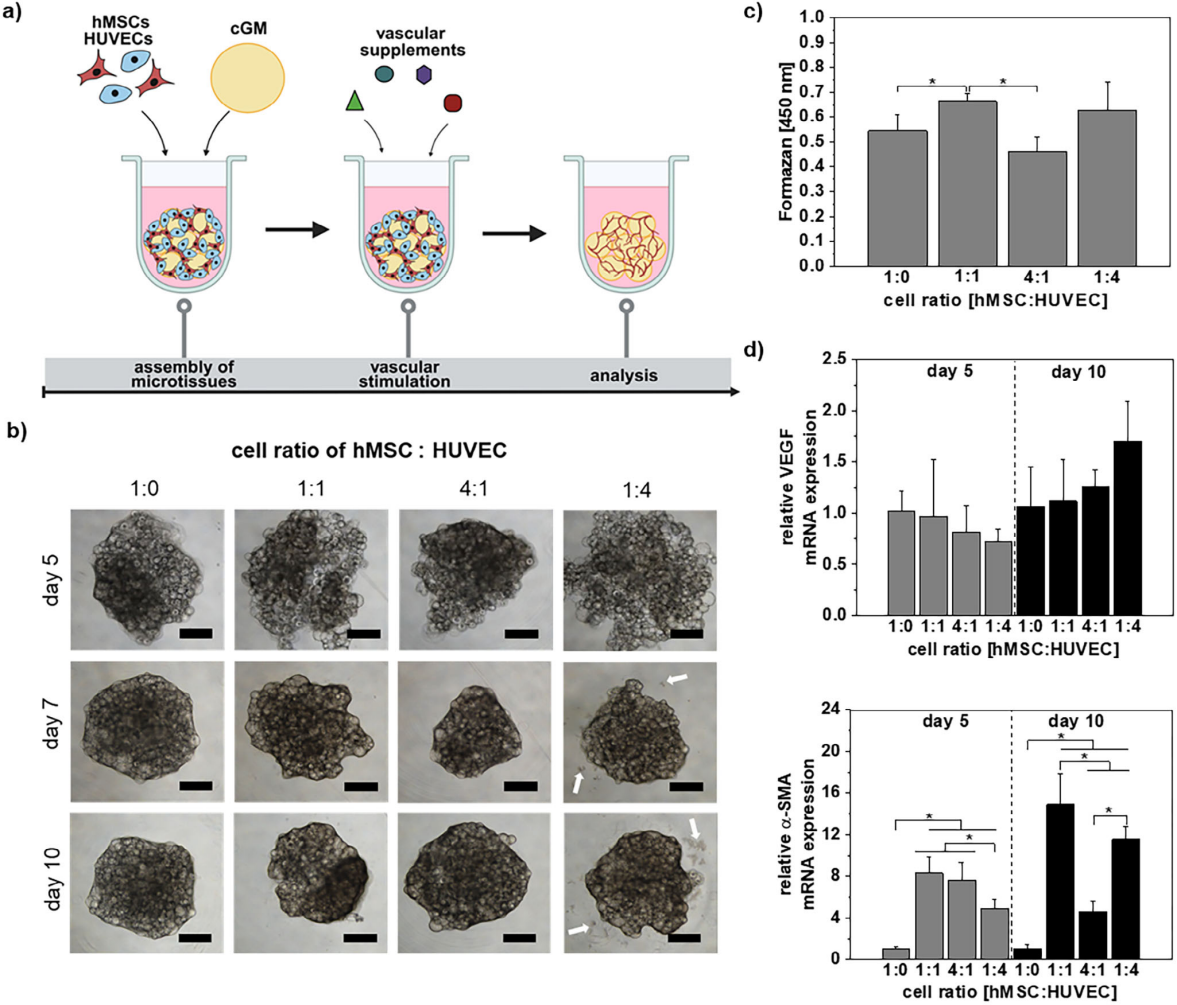
Effect of cGM as a cell adhesive material for the assembly of vascular microtissues to study effects of siRNA on osteogenesis‐angiogenesis interaction. a) Experimental set‐up: A co‐culture of hMSCs and HUVECs with a total of 10 000 cells/microtissues in varying cell ratios was aggregated with 0.128 mg crosslinked gelatin microparticles, and differentiation was induced by cultivation with vascular supplements. The hMSCs are supposed to serve as pericyte‐like cells to stabilize the formed vascular structures by HUVECs. Created in BioRender. Mitrach, F. (2025) b) Microscopical analysis showed the formation of uniform and stable microtissues independent of cell ratio but some detached cells from microtissues for 1:4 cell ratio (white arrows). c) Cell viability of vascular microtissues on day 10 measured via WST‐8 assay indicated higher cell viability of a hMSCs:HUVEC cell ratio of 1:1 than hMSC only or the 4:1 ratio. High standard deviations for the 1:4 ratio may result from detached cells. d) Gene expression levels of endothelial cell marker *VEGF* and pericyte marker *α‐SMA* were analyzed on days 5 and 10 of vascular differentiation via quantitative real‐time PCR. Results showed no significant influence of the cell ratio on expression of endothelial marker VEGF, but for pericyte marker α‐SMA highest expression was observed for a cell ratio 1:1 at both analysis days. Data are presented as mean ± SD (*n* = 4), two‐way ANOVA with Tukey's post hoc test. Statistically significant differences are indicated with (∗) between the different groups (*p* < 0.05). *α‐SMA*: alpha smooth muscle actin; *cGM*: cross‐linked gelatin microparticles; *hMSC*: human mesenchymal stem cells; *HUVECs*: human umbilical vein endothelial cells; *mRNA*: messenger RNA; *VEGF*: vascular endothelial growth factor.

Independent of the cell ratio, microscopical analysis showed the formation of uniform and stable vascular microtissues within 10 days, confirming the suitability of the gelatin‐based material as cell‐adhesive material for vascular cells in line with previous studies [56, 57]. However, for the cell ratio 1:4 (hMSC:HUVEC), a relevant number of cells was detected on the bottom of the well not associated with the microtissues (Figure 5b). Analysis of cell viability using WST‐8 assay showed highest cell viabilities for 1:1 and 1:4 ratios, whereas high standard deviations were observed for 1:4 ratio, obviously caused by incomplete adherence of cells to cGM/microtissues (Figure 5c). Gene expression analysis on days 5 and 10 revealed no significant influence of cell ratio on expression of endothelial marker *VEGF*, but significant differences were observed for pericyte marker α‐smooth muscle actin (*α‐SMA*), also indicating vessel maturation. Highest gene expression levels were observed for a cell ratio of 1:1 with a strong increase from day 5 to day 10. Lowest gene expression levels of *α‐SMA* were measured for microtissues without HUVECs (cell ratio 1:0), indicating successful interaction of hMSCs and HUVECs within microtissues with cGM as cell adhesive material suitable for formation of vascular microtissues (Figure 5d). Due to partial cell detachment observed for cell ratio 1:4, we decided to use a cell ratio of 1:1 for further experiments. This suggests that balanced hMSC–HUVEC ratios not only stabilize microtissues but also promote more mature vascular phenotypes, consistent with enhanced angiogenic signaling reported for 1:1 co‐cultures [32, 35, 56]

### 3.5 Interaction Between Osteogenic and Vascular Microtissues in Response to siRNA‐mediated Silencing of Chordin and WWP‐1

Having established stable and viable vascular microtissues through optimized co‐cultures of hMSCs and HUVECs with cGM as a cell‐adhesive scaffold, we next aimed to investigate the functional interaction between these vascular microtissues and the optimized osteogenic microtissues subjected to siRNA‐mediated silencing of Chordin and WWP‐1. For this purpose, pre‐differentiated osteogenic and vascular microtissues were combined within a fibrin hydrogel matrix, enabling direct interaction and allowing us to assess if and how siRNA‐mediated silencing influences vascular outgrowth and osteogenic mineralization.

#### 3.5.1 Microtissue Interaction in Response to Chordin siRNA Treatment

**FIGURE 6:**
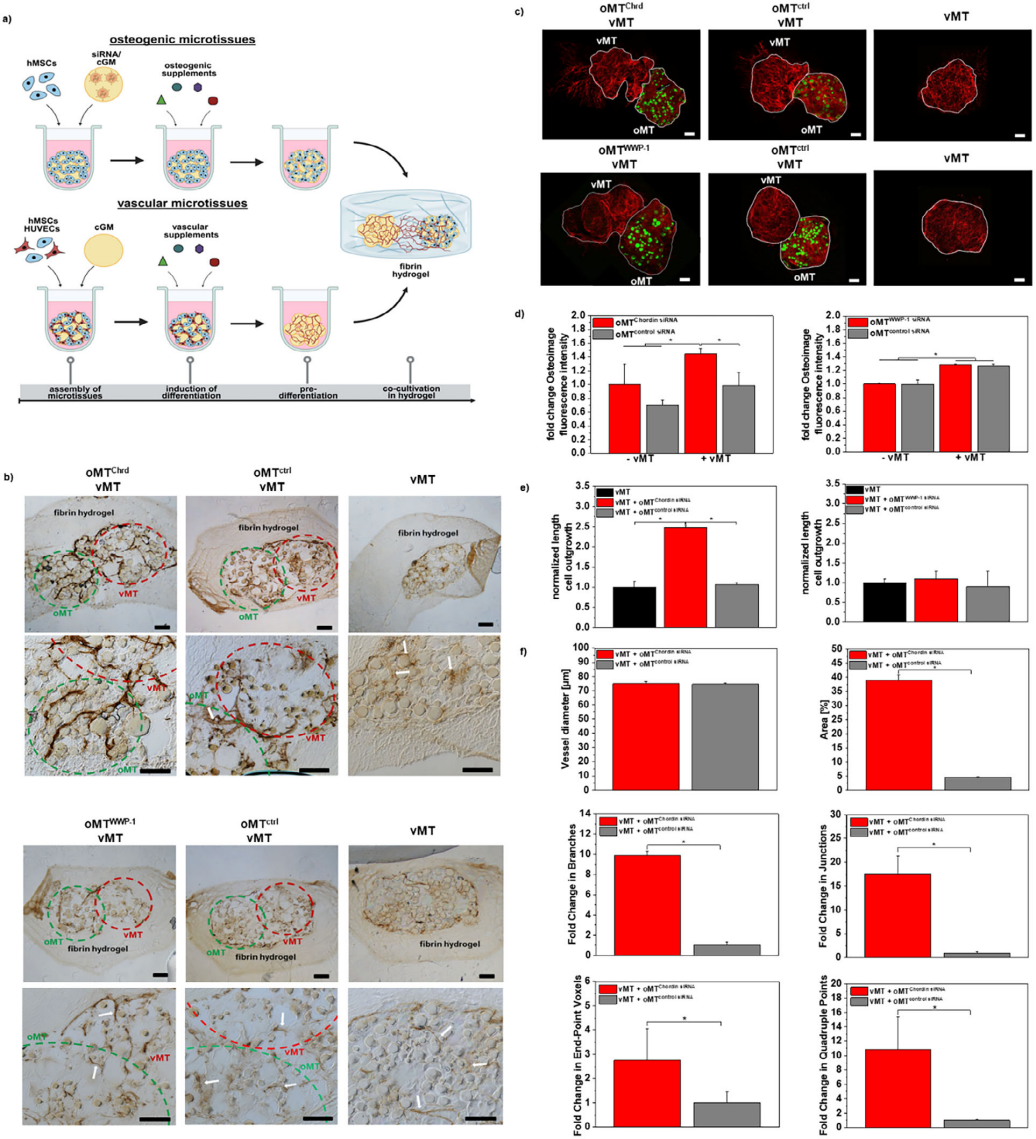
Analysis of interaction between osteogenic and vascular microtissues upon Chordin or WWP‐1 siRNA treatment of osteogenic microtissues. a) Experimental set‐up: Osteogenic and vascular microtissues were predifferentiated for 14 days in their respective differentiation environment to induce the appropriate lineage commitment. Afterwards, both microtissues were combined in a fibrin hydrogel and cultivated for up to 32 days. Created in BioRender. Mitrach, F. (2025) b) Immunohistochemical staining of cryosections for endothelial marker CD31/PECAM‐1. Positive cells appear as brown and are indicated by a white arrow. Scale: 100 µm. c) Representative confocal laser scanning microscopy of osteogenic and vascular microtissues embedded in a fibrin hydrogel. To visualize hydroxyapatite formation, Osteoimage mineralization assay was performed in combination with AlexaFluor 568 Phalloidin staining to visualize cell morphology and outgrowth of vascular structures into surrounding fibrin matrix. d) Quantification of fluorescence intensity of Osteoimage mineralization assay as a measure for hydroxyapatite formation was performed by using ImageJ on days 21 and 32 after combination of osteogenic and vascular microtissues. e) Measurement of distance of cell outgrowth from the edge of vascular microtissues into the surrounding fibrin matrix was done via ImageJ on day 32. f) Vascular network quantification for vMT co‐cultivated with oMT treated with Chordin siRNA or control siRNA at day 32 of co‐culture. Data were extracted from 3D fluorescence image stacks and analyzed with the Angiogenesis Analyzer and Analyze Skeleton plugins in ImageJ software (version 1.54, NIH, Bethesda, MD, USA). Data are presented as mean ± SD (*n* = 4). Statistically significant differences are indicated with (∗) between the different groups (*p* < 0.05), one‐way ANOVA with Tukey's post hoc test. *cGM*: cross‐linked gelatin microparticles; *hMSCs*: human mesenchymal stem cells; *HUVECs*: human umbilical vein endothelial cells; *oMT*: osteogenic microtissues; *siRNA*: small interfering RNA; *vMT*: vascular microtissues.

Microscopical examination showed that vascular microtissues alone or combined with osteogenic microtissues^ctrl^ only showed weak outgrowth of vascular structures. In contrast, the co‐culture of vascular microtissues with osteogenic microtissues^Chrd^ supported the outgrowth of vascular structures (Figure 6c). Based on this microscopic examination, we used ImageJ to measure the distance of cell outgrowth from the edge of vascular microtissues into the surrounding fibrin matrix. Cultivation of vascular microtissues with osteogenic microtissues^Chrd^ resulted in a 2.5‐fold increase in length of outgrowth vascular structures in comparison to vascular microtissues alone or vascular microtissues combined with osteogenic microtissues^ctrl^ (Figure 6e). Combining vascular microtissues with osteogenic microtissues^Chrd^ further revealed a stronger expression of CD31/PECAM‐1 in comparison to vascular microtissues alone or vascular microtissues combined with osteogenic microtissues^ctrl^. We found a clear ingrowth of vessel structures from vascular microtissues into osteogenic microtissues^Chrd^. In contrast, for vascular microtissues alone, only a weak CD31/PECAM‐1 staining without clearly distinguished structures was observed (Figure 6b).

Fluorescence intensity of Osteoimage mineralization assay as a measure for hydroxyapatite formation within hydrogel was also performed using ImageJ. The highest fluorescence intensities were found for osteogenic microtissues^Chrd^ combined with vascular microtissues. In contrast, osteogenic microtissues^Chrd^ and osteogenic microtissues^ctrl^ cultivated without vascular microtissues showed no significant differences for both analysis days (Figure 6e).

Vessel diameter remained unchanged upon Chordin silencing. Microtissues treated with Chordin siRNA displayed mean vessel diameters comparable to those observed under treatment with control siRNA (approximately 70‐75 µm; Figure 6f), with only minimal variation between groups. Consistent with this, statistical analysis did not detect any significant differences, indicating that siRNA‐mediated Chordin knockdown does not measurably affect vessel diameter under the applied culture conditions. In contrast to the unaffected vessel diameter, Chordin silencing in osteogenic microtissues had a pronounced impact on the extent of vascular outgrowth from vascular microtissues, which was assessed by quantifying vascular density as the ratio of outgrowing vascular structures to the total microtissue area. Quantitative image analysis revealed that Chordin silencing in osteogenic microtissues resulted in a nearly eight‐fold higher vessel area (approximately 40%; Figure 6f) compared to control siRNA conditions (approximately 4%; Figure 6f). This increase was statistically significant, indicating that Chordin silencing strongly promotes the outgrowth of vascular structures from vascular microtissues. We further found that increased interaction in response to siRNA‐mediated Chordin knockdown in microtissues significantly increased the complexity of the outgrown vascular network. Quantitative image analysis demonstrated a marked increase in morphometric parameters relative to control co‐cultures. Specifically, the number of quadruple points (junctions where four segments connecting) and junction voxels (points where two or more segments are interconnected) were strongly enhanced. In addition, end‐point voxels—defined as the terminal points at the ends of vessel segments—were more abundant, indicating increased numbers of newly initiating sprouts. Finally, the number of branches (linear vessel segments between network nodes) was increased, indicating expanded vascular outgrowth. Taken together, the results indicate that Chordin silencing enhances the formation of a denser, highly branched endothelial network within the co‐cultures. Furthermore, these findings demonstrate that siRNA‐mediated Chordin silencing in osteogenic microtissues strengthens the interaction between osteogenic and angiogenic compartments, highlighting the potential of local siRNA delivery to bridge the gap between osteogenesis and angiogenesis in the engineering of vascularized bone tissue constructs.

#### 3.5.2 No Microtissue Interaction in Response to WWP‐1 siRNA Treatment

Contrary to the results for Chordin siRNA, silencing of WWP‐1 had no considerable impact on the outgrowth of vascular structures. We observed no structures that grew out from the edge of the vascular microtissues into the surrounding hydrogel matrix in the presence or absence of WWP‐1 siRNA treatment (Figure 6c,e). Immunohistochemistry showed only a weak CD31/PECAM‐1 staining for all groups (Figure 6b).

Fluorescence intensity of Osteoimage mineralization assay as a measure for hydroxyapatite formation was quantified on day 32 after combination of osteogenic and vascular microtissues using ImageJ. Osteogenic microtissues cultivated with vascular microtissues showed a significant higher Osteoimage mineralization assay fluorescence intensity in comparison to osteogenic microtissues cultivated without vascular microtissues, indicating a proosteogenic cross‐talk between osteogenic and angiogenic microtissues. However, we found no significant influence of WWP‐1 silencing on hydroxyapatite formation (Figure 6e). Taken together, these results indicate that WWP‐1 siRNA treatment can lead to an earlier induction of osteogenic differentiation but shows no significant impact on interaction between osteogenic and angiogenic microtissues.

## 4 Discussion

siRNA‐mediated knockdown of negative regulators governing the osteogenesis–angiogenesis cross‐talk offers a promising strategy to stimulate vascularized bone formation. In earlier work, our group provided proof‐of‐concept evidence indicating that siRNA‐loading of cell‐adhesive cross‐linked gelatin microparticles (cGM) assembled with hMSCs to microtissues can effectively downregulate the BMP‐2 antagonist Chordin, thereby enhancing osteogenic differentiation [10, 11]. While this previous investigation utilized the commercial siRNA transfection reagent Lipofectamine RNAiMax, its constraints in terms of limited siRNA loading capacity and compatibility prompted us to explore alternative delivery systems. For this purpose, we applied oligomer‐stabilized calcium phosphate nanoparticles (CaP‐NP) [51] to achieve higher siRNA loading of cGM, aiming to increase transfection efficiency within microtissues.

Systematic analysis of the cGM loading with CaP‐NP formulation revealed that siRNA silencing efficiency critically depends on cGM cross‐linking degree and nanoparticle loading (Figure 2, Figures S1 and S2). While cross‐linking ensures particle stability for long‐term culture, excessive cross‐linking with the maleic anhydride oligomer induced crystal‐like CaP precipitates, likely due to local pH reduction, impairing nanoparticle integrity and transfection (Figure S1). A lower cross‐linking degree preserved CaP‐NP stability and markedly enhanced silencing (Figure 2). Increasing the CaP‐NP load further improved efficiency, achieving complete *Chordin* suppression (Figure 2). We believe that the strong silencing effect arises from sustained contact between CaP‐NP‐loaded cGM and adherent cells, enabling efficient uptake and avoiding NP sedimentation issues, which typically occur in 2D cultures. Supporting this, monolayer transfections showed markedly lower Chordin knockdown and osteogenic activity (Figure S4). Together, these findings underscore the superior performance of cGM‐based siRNA delivery in 3D microtissues as an advanced platform for gene modulation studies. Notably, loading of cGM with siRNA/CaP‐NP complexes outperformed siRNA/Lipofectamine complexes, which only achieved partial Chordin downregulation under otherwise identical experimental conditions [10, 11].

To investigate functional consequences of siRNA‐mediated silencing to orchestrate osteogenic‐vascular cross‐talk, we targeted two distinct antagonists: Chordin, an extracellular antagonist of BMP‐2 that blocks BMP‐2 signaling; and WWP‐1, an E3 ubiquitin ligase that mediates proteasomal degradation of osteogenic transcription factors.

In order to analyze the differentiation state and functionality of the *Chordin* and *WWP‐1* silenced microtissues on a broad scale, we performed next‐generation sequencing of osteogenic microtissues. For Chordin‐silenced microtissues, we observed a pronounced activation of both BMP and WNT signaling pathways. Within the BMP pathway, the key osteogenic transcription factor *RUNX2*, along with *BMP2*, *BMP4*, and the downstream mediators *SMAD1* and *SMAD9*, were markedly upregulated at day 14, highlighting a strong pro‐osteogenic response. Interestingly, inhibitory regulators of BMP signaling, including *SMURF1* – which ubiquitinates BMP downstream components – and *SMAD7*, were also upregulated, suggesting the presence of feedback mechanisms for the fine‐tuning of osteogenic activity. Canonical and noncanonical WNT signaling pathways were likewise enhanced, as indicated by elevated expression of *WNT5B*, *WNT10B*, and *WNT11*, together with *CTNNB1* (encoding β‐catenin) and the co‐receptor *LRP5*. In addition, *DMP1* (dentin matrix acidic phosphoprotein 1), a marker associated with late‐stage osteoblast differentiation and bone matrix formation, was upregulated, whereas *TNFSF11* (encoding RANKL), a key inducer of osteoclastogenesis, was downregulated. Together, these transcriptomic findings indicate a sustained osteogenic program that persisted beyond the period of Chordin silencing, underscoring a stable shift toward bone‐forming activity.

For the angiogenic mRNAs, we found upregulation of *HIF1A* (Hypoxia‐Inducible Factor 1‐Alpha), *CXCL5* and *CXCL6*, *VEGFB* (stimulates formation of new blood vessels) and *VEGFC* (supports lymphangiogenesis of lymphatic endothelial cells), *NRP2* (a co‐receptor for VEGF), *EDN1* (Endothelin), *ANGPT2* (Angiopoietin 2), *SERPINF1 –*(coding for Pigment Epithelium‐Derived Factor, a neurotrophic factor and inhibitor of angiogenesis), all with the exception of *SERPINF1* indicating angiogenic stimulation.

In *WWP‐1*‐silenced microtissues, both canonical and noncanonical WNT signaling pathways were upregulated, similar to the response observed in *Chordin*‐silenced microtissues. This was reflected by increased expression of *WNT5B*, *WNT10B*, *WNT11*, and the osteogenic transcription factor *RUNX2*. In contrast to the Chordin‐silenced group, however, components of the BMP signaling pathway – specifically *SMAD5*, *BMPR1B*, *BGLAP* (osteocalcin), and *COL10A1* – were upregulated, indicating an additional activation of downstream osteogenic processes. Notably, negative regulators of bone formation such as *GSK3B*, which promotes β‐catenin degradation, and *TNFSF11* (encoding RANKL) also showed increased expression, suggesting a dynamic balance between bone formation and resorption.

The angiogenic gene expression profile showed parallels to the *Chordin*‐silenced condition, with transient upregulation of *HIF1A*, *CXCL5*, *EDN1*, *ANGPT2*, and *VEGFB*. However, unlike the Chordin‐silenced group where angiogenic signaling persisted to day 14, these factors were primarily upregulated at day 7 and declined thereafter. Moreover, *VEGFC* and *VEGFD* were consistently downregulated compared to non‐silenced controls across both time points.

Overall, WWP‐1 silencing triggered an early but transient angiogenic response and a complex osteogenic signaling pattern, characterized by both promotive and inhibitory influences on bone formation, distinct from the sustained osteogenic and angiogenic stimulation observed after Chordin silencing.

Functional relevance, however, can only be assessed by evaluating downstream biological responses. In order to investigate the effects of silencing osteogenic inhibitors on the tightly coupled osteogenesis and angiogenesis processes in bone regeneration, we set up a co‐culture of osteogenic and vascular microtissues, surpassing prior studies that have largely relied on simplified 2D monocultures or isolated assays serving to test conditioned media [58, 59, 60, 61]. To this end, we combined osteogenic and vascular microtissues within a fibrin hydrogel matrix, allowing us to assess whether siRNA‐mediated modulation of osteogenic microtissues influences their interaction with vascular microtissues. In agreement with the upregulation of angiogenic genes found in NGS, Chordin silencing markedly enhanced the angiogenic response, as reflected by increased outgrowth and ingrowth of structures from vascular microtissues but also clearly higher number of branches and junctions in the prevascularizing structures than in the control siRNA group (Figure 5b,d,e). Obviously, siRNA‐mediated silencing of Chordin enhanced osteogenesis‐angiogenesis crosstalk by alleviating its antagonism of BMP‐2. Consequently, BMP‐2 activity increased, which promoted osteogenic differentiation of mesenchymal cells and simultaneously upregulated angiogenic genes and VEGF secretion [62, 63, 64]. The secreted VEGF facilitated endothelial cell migration and tubule formation, while endothelial‐derived VEGF reciprocally enhanced osteogenic maturation through the BMP‐2/SMAD pathway [62, 63, 64]. This bidirectional signaling created a synergistic feedback loop coordinating matrix mineralization with vascular network integration in the microtissue [62, 63, 64]. To our knowledge, this is the first demonstration that Chordin silencing in osteogenic microtissues directly promotes their interaction with vascular microtissues within a co‐culture model.

In contrast, WWP‐1 downregulation did not elicit similar functional effects. Despite moderate silencing, osteogenic microtissues showed upregulation of angiogenic genes, but this effect did not remain until day 14. In agreement with the transcriptomic data, *WWP‐1*‐silenced microtissues did not secrete VEGF, nor did we observe vascular outgrowth or ingrowth (Figure 3e and 5b,d,e). WWP‐1, an E3 ubiquitin ligase targeting transcription factors like RUNX2 for degradation, was reported to mainly affect intracellular protein stability and signaling turnover rather than induction of signaling pathways [65, 66]. However, in our study we observed improved osteogenic differentiation, mainly by induction of the WNT‐signaling pathways, while the angiogenic stimulation may not have been consistent enough to induce vascular outgrowth. This mechanistic difference likely explains the lack of angiogenic response upon WWP‐1 silencing [65, 66]. Comparing these two antagonists, it is evident that not all osteogenic regulators equally influence osteogenic‐vascular paracrine crosstalk. While Chordin suppression stimulated the BMP and WNT signaling pathways, robustly reinforcing the BMP‐2/VEGF axis, and coupling osteogenesis and angiogenesis effectively, WWP‐1 silencing acts on intracellular signaling dynamics without amplifying vascular interactions. These insights underline the importance of careful target selection in siRNA‐based regenerative strategies to enhance vascularized bone tissue formation.

These findings make clear that a regulator's function in osteogenesis alone is not a reliable predictor of its impact on angiogenic interaction. In this regard, our results also emphasize the necessity of establishing the presented co‐culture model as a powerful platform to screen different siRNAs for their capacity to modulate osteogenesis–angiogenesis cross‐talk. Effective modulation of bone regeneration requires assessing molecular targets within the full complexity of a multicellular environment. Therefore, the development of dedicated screening platforms is crucial to systematically uncover regulators that not only drive osteogenesis but also actively enhance angiogenesis, thereby addressing both pillars of coordinated tissue regeneration. Having established the utility of our co‐culture model for evaluating specific antagonists, an important next step will be to broaden its application to a wider range of molecular targets and signaling pathways [15, 67]. Nevertheless, it must be emphasized that the current system is particularly suited for the study of negative regulators that act during the early phases of osteogenic differentiation. This restriction arises from two key factors: first, the assembly of cells with the siRNA‐loaded cGM prevents subsequent transfection steps during later osteogenic differentiation, and second, siRNA exerts a temporary silencing effect because its intracellular presence and activity diminish over time. Together, these factors confine the applicability of our approach to early‐acting antagonists, while regulators expressed only at later stages cannot be effectively addressed.

Another important consideration in siRNA‐based approaches is the varying expression levels of individual inhibitory regulators, which significantly influences RNA interference efficiency [68, 69]. When comparing the knockdown efficiency of *Chordin* and *WWP‐1*, clear differences emerged between the two targets. While siRNA treatment of osteogenic microtissues against Chordin led to efficient target suppression, *WWP‐1* silencing was less pronounced, achieving only about 40% reduction in mRNA expression compared to control groups (Figure 3b). These discrepancies can be better understood by considering their endogenous expression profiles. Threshold cycle analysis revealed that *WWP‐1* is transcribed at markedly higher levels (Ct: 23) than Chordin (Ct: 33). When a target mRNA is highly abundant, it can overwhelm the RNA‐induced silencing complex (RISC), thereby limiting the maximum achievable gene knockdown [69, 70, 71]. This explains why *WWP‐1* exhibited comparatively lower silencing with our siRNA delivery platform. Increasing siRNA concentration raises the probability of RISC loading, thus improving silencing efficiency [69, 70, 71]. Additionally, chemical modifications such as 2′‐O‐methyl and 2′‐fluoro substitutions would strengthen siRNA stability, binding affinity, and promote efficient incorporation into RISC [3, 72, 73]. These modifications could also reduce degradation and off‐target effects, extending siRNA functional lifespan in biological environments [3, 72, 73]. Moreover, chemically stabilized siRNA may also address target mRNAs that are expressed later during osteogenic differentiation, which is not possible with our current approach. With the idea to later employ microtissues as building blocks for regenerative applications, we intended, however, a limited presence of siRNA in microtissues that would not be available anymore beyond day 10. This strategy prevents issues of unwanted siRNA effects via siRNA escape from microtissues after implantation.

Nevertheless, the siRNA delivery approach implemented in this study offers a high degree of versatility, since only the siRNA sequence needs to be adjusted to silence alternative antagonists.

The adaptability of our 3D co‐culture microtissue platform is especially valuable in the complex context of bone regeneration, where multiple inhibitory pathways may act simultaneously or in a stage‐dependent manner. This platform offers a flexible experimental environment for systematic screening by allowing comparative evaluation of different transfection reagents, siRNA sequences, and chemical modifications. It also supports the use of primary cells from diverse donor sources, enabling capture of inter‐donor variability in regenerative responses. Since the efficacy of siRNA modulation can vary significantly with donor factors such as gender, age, and health status, our system, although standardized on healthy young donor cells for consistency, can be extended to include patient‐derived cells (e.g., from individuals with osteoporosis or diabetes) and gender‐specific sources. This capability allows a systematic investigation of how disease states and donor‐related variables influence siRNA regulation and bone regeneration outcomes. Such flexibility facilitates personalized regenerative medicine by enabling targeted screening of therapeutic siRNA candidates in biologically relevant and disease‐specific contexts.

In this way, our platform is not limited to testing a single antagonist but can be expanded to investigate a broad range of different antagonists of osteogenic‐angiogenic cross‐talk.

Additionally, we were able to show that our co‐cultures of osteogenic microtissues^Chrd^ and vascular microtissues with cGM as cell‐adhesive substrate were able to preserve stable vascular structures for at least 32 days. This longevity substantially exceeds what is typically reported for comparable in vitro co‐culture systems, where vascular structures often regress or lose integrity within shorter time frames of ∼14 days [32, 57, 74, 75, 76]. Such sustained stability indicates that the increased osteogenic microenvironment not only supports vascular maintenance but also may provide conditions that protect vessel integrity over extended periods. These findings emphasize the robustness of our advanced co‐culture platform and open opportunities to investigate long‐term osteogenesis‐angiogenesis cross‐talk under controlled conditions that more closely reflect the temporal dimension of bone healing.

Despite these advances over conventional angiogenesis assays, some limitations persist and need to be addressed. While the platform maintains sustained microtissue viability and function for 32 days – demonstrating exceptional long‐term culture stability uncommon in most in vitro models – challenges in scalability and translational relevance persist [32, 57, 74, 75, 76]. Manual procedures for microparticle loading and microtissue formation limit throughput and introduce batch‐to‐batch variability, hindering reproducibility for high‐throughput or large‐scale applications [26, 77, 78]. Emerging automated biofabrication and bioprinting technologies address these limitations by enabling precise, reproducible assembly with high cell viability and spatial control [26, 77, 78]. Specifically, liquid‐handling robots can automate siRNA‐nanoparticle loading onto cross‐linked gelatin microparticles (cGMs), followed by sterile mixing with cell suspensions [79, 80, 81]. Subsequent 3D bioprinting facilitates layer‐by‐layer microtissue association, while modular robotic systems ensure accurate positioning into complex constructs [79, 80, 81]. This automation may reduce manual variability, streamline workflows, and support standardized quality control for scalable production of vascularized bone models [79, 80, 81].

The widespread use of HUVECs in angiogenic research ensures standardization and comparability across studies, however, it is important to recognize that they may not adequately represent the functional heterogeneity of primary endothelial cells in vivo [34, 82, 83]. Similarly, while fibrin hydrogels offer biocompatibility and natural cell adhesion motifs, their rapid degradation and limited mechanical robustness constrain long‐term applications [84, 85, 86]. Hydrogels based on collagen or gelatin have gained particular interest as they not only supply native extracellular matrix components but also enable tailored biomechanical modifications to better support cellular functions [87, 88]. Notably, current in vitro microtissue models, including this platform, face intrinsic limitations in fully recapitulating chronic disease conditions. The complexity and gradual progression of pathophysiological processes such as osteoporosis, which evolve over months or years and involve multiple interacting cell types including osteoblasts, osteoclasts, and neuronal cells, pose significant challenges for in vitro modelling and may thus limit the predictive accuracy for chronic disease states. Despite these challenges, this platform holds considerable potential for advancing regenerative therapies aimed at restoring vascularized bone and tissue function in chronic diseases such as diabetes and osteoporosis. By leveraging donor‐derived cells and incorporating disease‐relevant biochemical stimuli, the platform offers a versatile and powerful approach to identify promising biomaterials or siRNA candidates, facilitating the development of autologous, personalized therapies to enhance tissue repair during disease‐related conditions.

## 5 Conclusion

To conclude, this study establishes a modular human co‐culture platform as a physiologically relevant model to evaluate siRNA impact on osteogenic–vascular cross‐talk. Employing a box‐in a box‐system with highly concentrated siRNA loaded CaP‐NP in cell‐adhesive cGMs as local siRNA carriers and delivery system, we were able to generate predifferentiated microtissues after assembling cGMs with hMSCs. NGS analysis confirmed that siRNA‐mediated silencing of negative regulators, such as Chordin or WWP‐1, successfully induced BMP and WNT signaling pathways as well as angiogenic stimulators in the case of Chordin silencing. These microtissues were combined with vascular microtissues consisting of HUVECs, hMSC and cGMs in order to set up a functional test system for siRNA effects on the osteogenic‐vascular cross‐talk. This system overcomes key limitations of both, conventional angiogenesis assays and inhomogeneous siRNA transfection in 3D cell cultures. Targeted silencing of negative regulators within this platform enables precise modulation of multicellular signaling pathways, providing critical insight into siRNA‐regulated mechanisms of vascularized bone formation. In future studies, siRNA targets could be tailored to address individual patient needs, paving the way for personalized microtissues as modular building blocks for bone regeneration therapies.

## Author Contributions

F.M.–conceptualization, investigation, original draft writing, funding acquisition; J.K.–investigation; S.S.–investigation; A.H.S.–investigation; A.L.–investigation; B.D.–investigation; M.C.H.–conceptualization, writing – review & editing, supervision; M.S.–conceptualization, writing – review & editing, supervision, funding acquisition. All authors have read and agreed to the published version of the manuscript.

## Funding

This project is funded by the junior research grant of the medical faculty of Leipzig and the Roland‐Ernst‐Stiftung für Gesundheitswesen.

## Conflicts of Interest

The authors declare that they have no known competing financial interests or personal relationships that could have appeared to influence the work reported in this paper.

## Supporting information




**Supporting File**: dhm70789‐sup‐0001‐SuppMat.pdf.

## Data Availability

The data that support the findings of this study are available from the corresponding author upon reasonable request.

## References

[1] S. Naeem , J. Zhang , Y. Zhang , and Y. Wang , “Nucleic Acid Therapeutics: Past, Present, and Future,” Molecular Therapy Nucleic Acids 36 (2025): 102440, 10.1016/j.omtn.2024.102440. 39897578 PMC11786870

[2] X. Sun , S. Setrerrahmane , C. Li , J. Hu , and H. Xu , “Nucleic Acid Drugs: Recent Progress and Future Perspectives,” Signal Transduction and Targeted Therapy 9 (2024): 316, 10.1038/s41392-024-02035-4. 39609384 PMC11604671

[3] B. Hu , L. Zhong , Y. Weng , et al., “Therapeutic siRNA: State of the Art,” Signal Transduction and Targeted Therapy 5 (2020): 101, 10.1038/s41392-020-0207-x. 32561705 PMC7305320

[4] W. Alshaer , H. Zureigat , A. Al Karaki , et al., “siRNA: Mechanism of Action, Challenges, and Therapeutic Approaches,” European Journal of Pharmacology 905 (2021): 174178, 10.1016/j.ejphar.2021.174178. 34044011

[5] S. M. Sarett , C. E. Nelson , and C. L. Duvall , “Technologies for Controlled, Local Delivery of siRNA,” Journal of Controlled Release 218 (2015): 94–113, 10.1016/j.jconrel.2015.09.066. 26476177 PMC4665980

[6] T. Tang , Y. Deng , J. Chen , et al., “Local Administration of siRNA through Microneedle: Optimization, Bio‐distribution, Tumor Suppression and Toxicity,” Scientific Reports 6 (2016): 30430, 10.1038/srep30430. 27457182 PMC4960650

[7] N. Lv , Z. Zhou , M. Hou , et al., “Research Progress of Vascularization Strategies of Tissue‐engineered Bone,” Frontiers in Bioengineering and Biotechnology 11 (2023): 1291969, 10.3389/fbioe.2023.1291969. 38312513 PMC10834685

[8] A. Shakeel and P. R. Corridon , “Mitigating Challenges and Expanding the Future of Vascular Tissue Engineering—Are We There yet?,” Frontiers in Physiology 13 (2022): 1079421, 10.3389/fphys.2022.1079421. 36685187 PMC9846051

[9] H. Liu , H. Chen , Q. Han , et al., “Recent Advancement in Vascularized Tissue‐engineered Bone Based on Materials Design and Modification,” Materials Today Bio 23 (2023): 100858, 10.1016/j.mtbio.2023.100858. PMC10679779 38024843

[10] S. Hinkelmann , A. H. Springwald , S. Schulze , et al., “Mineralizing Gelatin Microparticles as Cell Carrier and Drug Delivery System for siRNA for Bone Tissue Engineering,” Pharmaceutics 14 (2022): 548, 10.3390/pharmaceutics14030548. 35335924 PMC8949427

[11] S. Hinkelmann , A. H. Springwald , A. Starke , et al., “Microtissues from Mesenchymal Stem Cells and siRNA‐loaded Cross‐linked Gelatin Microparticles for Bone Regeneration,” Materials Today Bio 13 (2022): 100190, 10.1016/j.mtbio.2021.100190. PMC8693629 34988418

[12] A. Grosso , A. Lunger , M. G. Burger , et al., “VEGF Dose Controls the Coupling of Angiogenesis and Osteogenesis in Engineered Bone,” npj Regenerative Medicine 8 (2023): 15, 10.1038/s41536-023-00288-1. 36914692 PMC10011536

[13] M. G. Burger , A. Grosso , P. S. Briquez , et al., “Robust Coupling of Angiogenesis and Osteogenesis by VEGF‐decorated Matrices for Bone Regeneration,” Acta Biomaterialia 149 (2022): 111–125, 10.1016/j.actbio.2022.07.014. 35835287

[14] T. Genova , S. Petrillo , E. Zicola , et al., “The Crosstalk between Osteodifferentiating Stem Cells and Endothelial Cells Promotes Angiogenesis and Bone Formation,” Frontiers in Physiology 10 (2019): 1291, 10.3389/fphys.2019.01291. 31681005 PMC6802576

[15] S. Li , X. Cai , J. Guo , et al., “Cell Communication and Relevant Signaling Pathways in Osteogenesis–angiogenesis Coupling,” Bone Research 13 (2025): 45, 10.1038/s41413-025-00417-0. 40195313 PMC11977258

[16] S. Damaraju , J. R. Matyas , D. E. Rancourt , and N. A. Duncan , “The Role of Gap Junctions and Mechanical Loading on Mineral Formation in a Collagen‐I Scaffold Seeded with Osteoprogenitor Cells,” Tissue Engineering Part A 21 (2015): 1720–1732, 10.1089/ten.tea.2014.0522. 25752490 PMC4426311

[17] C. Wang , S. Stöckl , S. Li , et al., “Effects of Extracellular Vesicles from Osteogenic Differentiated Human BMSCs on Osteogenic and Adipogenic Differentiation Capacity of Naïve Human BMSCs,” Cells 11 (2022), 10.3390/cells11162491. PMC9406723 36010568

[18] N. Al‐Sharabi , S. Mohamed‐Ahmed , S. Shanbhag , et al., “Osteogenic human MSC‐derived Extracellular Vesicles Regulate MSC Activity and Osteogenic Differentiation and Promote Bone Regeneration in a Rat Calvarial Defect Model,” Stem Cell Research & Therapy 15 (2024): 33, 10.1186/s13287-024-03639-x. 38321490 PMC10848378

[19] G. Basha , A. G. Cottle , T. Pretheeban , et al., “Lipid Nanoparticle‐mediated Silencing of Osteogenic Suppressor GNAS Leads to Osteogenic Differentiation of Mesenchymal Stem Cells in Vivo,” Molecular Therapy 30 (2022): 3034–3051, 10.1016/j.ymthe.2022.06.012. 35733339 PMC9481989

[20] Y. Wang , M. Liu , W. Zhang , et al., “Mechanical Strategies to Promote Vascularization for Tissue Engineering and Regenerative Medicine,” Burns & Trauma 12 (2024): tkae039, 10.1093/burnst/tkae039. 39350780 PMC11441985

[21] F. Wu , C. Song , G. Zhen , et al., “Exosomes Derived from BMSCs in Osteogenic Differentiation Promote Type H Blood Vessel Angiogenesis through miR‐150‐5p Mediated Metabolic Reprogramming of Endothelial Cells,” Cellular and Molecular Life Sciences 81 (2024): 344, 10.1007/s00018-024-05371-4. 39133273 PMC11335269

[22] W. He , X. Shi , Z. Guo , H. Wang , M. Kang , and Z. Lv , “Circ_0019693 promotes Osteogenic Differentiation of Bone Marrow Mesenchymal Stem Cell and Enhances Osteogenesis‐coupled Angiogenesis via Regulating microRNA‐942‐5p‐targeted Purkinje Cell Protein 4 in the Development of Osteoporosis,” Bioengineered 13 (2022): 2181–2193, 10.1080/21655979.2021.2023982. 35030971 PMC8973649

[23] Q. Chang , M. Fujio , M. Tsuboi , H. Bian , M. Wakasugi , and H. Hibi , “High‐mobility Group Box 1 Accelerates Distraction Osteogenesis Healing via the Recruitment of Endogenous Stem/Progenitor Cells,” Cytotherapy 25 (2023): 946–955, 10.1016/j.jcyt.2023.05.013. 37354151

[24] D. Wu , L. Liu , S. Fu , and J. Zhang , “Osteostatin Improves the Osteogenic Differentiation of Mesenchymal Stem Cells and Enhances Angiogenesis through HIF‐1α under Hypoxia Conditions in Vitro,” Biochemical and Biophysical Research Communications 606 (2022): 100–107, 10.1016/j.bbrc.2022.02.085. 35339748

[25] Y. Kang , T. H. Kang , H. S. Ro , N. S. Hwang , and H. D. Kim , “Engineering Osteogenic Spheroids: the Impact of Endothelial Cell Localization on Vascularization and Differentiation,” Advanced Healthcare Materials 14 (2025): 2501390, 10.1002/adhm.202501390. 40500989

[26] M. H. Kim , Y. P. Singh , N. Celik , et al., “High‐throughput Bioprinting of Spheroids for Scalable Tissue Fabrication,” Nature Communications 15 (2024): 10083, 10.1038/s41467-024-54504-7. PMC11582690 39572584

[27] G. Turnbull , J. Clarke , F. Picard , et al., “3D bioactive Composite Scaffolds for Bone Tissue Engineering,” Bioact Mater 3 (2018): 278–314, 10.1016/j.bioactmat.2017.10.001. 29744467 PMC5935790

[28] C. Correia , W. L. Grayson , M. Park , et al., “In Vitro Model of Vascularized Bone: Synergizing Vascular Development and Osteogenesis,” PLoS One 6 (2011): 28352, 10.1371/journal.pone.0028352. PMC3229596 22164277

[29] C. Correia , W. Grayson , R. Eton , et al., “Human Adipose‐derived Cells Can Serve as a Single‐cell Source for the in Vitro Cultivation of Vascularized Bone Grafts,” Journal of Tissue Engineering and Regenerative Medicine 8 (2014): 629–639, 10.1002/term.1564. 22903929 PMC4129644

[30] D. L. Hutton , E. M. Moore , J. M. Gimble , and W. L. Grayson , “Platelet‐derived Growth Factor and Spatiotemporal Cues Induce Development of Vascularized Bone Tissue by Adipose‐derived Stem Cells,” Tissue Engineering Part A 19 (2013): 2076–2086, 10.1089/ten.tea.2012.0752. 23582144 PMC3725877

[31] N. G. Schott and J. P. Stegemann , “Coculture of Endothelial and Stromal Cells to Promote Concurrent Osteogenesis and Vasculogenesis,” Tissue Engineering Part A 27 (2021): 1376–1386, 10.1089/ten.TEA.2020.0330. 33599160 PMC8827126

[32] N. G. Schott , H. Vu , and J. P. Stegemann , “Multimodular Vascularized Bone Construct Comprised of Vasculogenic and Osteogenic Microtissues,” Biotechnology and Bioengineering 119 (2022): 3284–3296, 10.1002/bit.28201. 35922969 PMC9547967

[33] F. Duttenhoefer , R. Lara de Freitas , T. Meury , et al., “3D scaffolds co‐seeded with human Endothelial Progenitor and Mesenchymal Stem Cells: Evidence of Prevascularisation within 7 Days,” European Cells and Materials 26 (2013): 49–64, 10.22203/ecm.v026a04. 23986333

[34] I. Kocherova , A. Bryja , P. Mozdziak , et al., “Human Umbilical Vein Endothelial Cells (HUVECs) Co‐Culture with Osteogenic Cells: from Molecular Communication to Engineering Prevascularised Bone Grafts,” Journal of Clinical Medicine 8 (2019): 1602, 10.3390/jcm8101602. 31623330 PMC6832897

[35] S. Zhang , M. Zhou , Z. Ye , Y. Zhou , and W.‐S. Tan , “Fabrication of Viable and Functional Pre‐Vascularized Modular Bone Tissues by Coculturing MSCs and HUVECs on Microcarriers in Spinner Flasks,” Biotechnology Journal 12 (2017), 10.1002/biot.201700008. 28544815

[36] K. Wirsig , N. Bürger , L. Fleischhauer , N. L. Preuß , and A. Bernhardt , “Vascularized in Vitro Bone Model as 3D Quadruple Culture with Primary human Osteoblasts, Osteocytes, Osteoclasts and Endothelial Cells,” Materials Today Bio 34 (2025): 102154, 10.1016/j.mtbio.2025.102154. PMC12345344 40809344

[37] K. Kellner , G. Liebsch , I. Klimant , et al., “Determination of Oxygen Gradients in Engineered Tissue Using a Fluorescent Sensor,” Biotechnology and Bioengineering 80 (2002): 73–83, 10.1002/bit.10352. 12209788

[38] S. Pelofy , H. Bousquet , L. Gibot , M.‐P. Rols , and M. Golzio , “Transfer of Small Interfering RNA by Electropermeabilization in Tumor Spheroids,” Bioelectrochemistry 141 (2021): 107848, 10.1016/j.bioelechem.2021.107848. 34118554

[39] K. A. Whitehead , R. Langer , and D. G. Anderson , “Knocking down Barriers: Advances in siRNA Delivery,” Nature Reviews Drug Discovery 8 (2009): 129–138, 10.1038/nrd2742. 19180106 PMC7097568

[40] Y. Gao , M. Li , B. Chen , et al., “Predictive Models of Diffusive Nanoparticle Transport in 3‐dimensional Tumor Cell Spheroids,” The AAPS Journal 15 (2013): 816–831, 10.1208/s12248-013-9478-2. 23605950 PMC3691442

[41] P. Cybulski , M. Bravo , J. J.‐K. Chen , et al., “Nanoparticle Accumulation and Penetration in 3D Tumor Models: the Effect of Size, Shape, and Surface Charge,” Frontiers in Cell and Developmental Biology 12 (2024): 1520078, 10.3389/fcell.2024.1520078. 39925825 PMC11802510

[42] S. Grebenyuk , A. R. Abdel Fattah , M. Kumar , et al., “Large‐scale Perfused Tissues via Synthetic 3D Soft Microfluidics,” Nature Communications 14 (2023): 193, 10.1038/s41467-022-35619-1. PMC9837048 36635264

[43] E. D. Bonifácio , C. A. Araújo , M. V. Guimarães , et al., “Computational Model of the Cancer Necrotic Core Formation in a Tumor‐on‐a‐chip Device,” Journal of Theoretical Biology 592 (2024): 111893, 10.1016/j.jtbi.2024.111893. 38944380

[44] O. Piwocka , K. Sterzyńska , A. Malińska , W. M. Suchorska , and K. Kulcenty , “Development of Tetraculture Spheroids as a Versatile 3D Model for Personalized Breast Cancer Research,” (2025), https://www.nature.com/articles/s41598‐025‐12556‐9?fromPaywallRec=false, (accessed 9 September 2025). 10.1038/s41598-025-12556-9 PMC12304226 40721466

[45] S. Lu , E. J. Lee , J. Lam , Y. Tabata , and A. G. Mikos , “Evaluation of Gelatin Microparticles as Adherent‐Substrates for Mesenchymal Stem Cells in a Hydrogel Composite,” Annals of biomedical engineering 44 (2016): 1894–1907, 10.1007/s10439-016-1582-x. 26935924 PMC4880535

[46] A. Vaziri , R. Maia , P. Zhang , et al., “Granular Hydrogels as Modular Biomaterials: from Structural Design to Biological Responses,” Advanced Healthcare Materials (2025): 02462, 10.1002/adhm.202502462. PMC12817117 41013958

[47] A. Joshi , A. Agrawal , S. Choudhury , et al., “From Microparticles to Bulk Hydrogels: Emerging Granular Hydrogels in Cartilage Tissue Engineering,” Biomaterials Science 13 (2025): 4916–4951, 10.1039/D5BM00801H. 40765258

[48] J. Bader , P. Rüedi , V. Mantella , et al., “Loading of Extracellular Vesicles with Nucleic Acids via Hybridization with Non‐Lamellar Liquid Crystalline Lipid Nanoparticles,” Advanced Science 12 (2025): 2404860, 10.1002/advs.202404860. 39741121 PMC11848734

[49] J. Bader , F. Brigger , and J.‐C. Leroux , “Extracellular Vesicles versus Lipid Nanoparticles for the Delivery of Nucleic Acids,” Advanced Drug Delivery Reviews 215 (2024): 115461, 10.1016/j.addr.2024.115461. 39490384

[50] J. Roerig , F. Mitrach , M. Schmid , et al., “Synergistic siRNA Loading of Extracellular Vesicles Enables Functional Delivery into Cells,” Small Methods 6 (2022): 2201001, 10.1002/smtd.202201001. 36284470

[51] F. Mitrach , M. Schmid , M. Toussaint , et al., “Amphiphilic Anionic Oligomer‐Stabilized Calcium Phosphate Nanoparticles with Prospects in siRNA Delivery via Convection‐Enhanced Delivery,” Pharmaceutics 14 (2022): 326, 10.3390/pharmaceutics14020326. 35214058 PMC8877163

[52] M. I. Love , W. Huber , and S. Anders , “Moderated Estimation of Fold Change and Dispersion for RNA‐seq Data with DESeq2,” Genome Biology 15 (2014): 550, 10.1186/s13059-014-0550-8. 25516281 PMC4302049

[53] Z. Gu , “Complex Heatmap Visualization,” iMeta 1 (2022): 43, 10.1002/imt2.43. PMC10989952 38868715

[54] F. Pedregosa , G. Varoquaux , A. Gramfort , et al., “Scikit‐learn: Machine Learning in Python,” Journal of Machine Learning Research 12 (2011): 2825–2830.

[55] H. Kibirige , G. Lamp , J. Katins , G. austin , and F. Finkernagel , “has2k1/plotnine: V0.14.5,” Zenodo (2025).

[56] M. Keshavarz and Q. Smith , “Gelatin‐Mediated Vascular Self‐Assembly via a YAP‐MMP Signaling Axis,” Advanced Functional Materials 34 (2024): 2402360, 10.1002/adfm.202402360. 39583865 PMC11583535

[57] J. Liu , Y. J. Chuah , J. Fu , W. Zhu , and D.‐A. Wang , “Co‐culture of human Umbilical Vein Endothelial Cells and human Bone Marrow Stromal Cells into a Micro‐cavitary Gelatin‐methacrylate Hydrogel System to Enhance Angiogenesis,” Materials Science and Engineering: C 102 (2019): 906–916, 10.1016/j.msec.2019.04.089. 31147062

[58] H. Schneider , B. Sedaghati , A. Naumann , M. C. Hacker , and M. Schulz‐Siegmund , “Gene Silencing of Chordin Improves BMP‐2 Effects on Osteogenic Differentiation of human Adipose Tissue‐derived Stromal Cells,” Tissue Engineering Part A 20 (2014): 335–345, 10.1089/ten.TEA.2012.0563. 23931154

[59] C. Wang , F. Xiao , Y. Gan , et al., “Improving Bone Regeneration Using Chordin siRNA Delivered by pH‐Responsive and Non‐Toxic Polyspermine Imidazole‐4,5‐Imine,” Cellular Physiology and Biochemistry 46 (2018): 133–147, 10.1159/000488416. 29587276

[60] C. Wang , W. Yuan , F. Xiao , et al., “Biscarbamate Cross‐Linked Low‐Molecular‐Weight Polyethylenimine for Delivering Anti‐chordin siRNA into Human Mesenchymal Stem Cells for Improving Bone Regeneration,” Frontiers in Pharmacology 8 (2017): 572, 10.3389/fphar.2017.00572. 28970797 PMC5609535

[61] F. N. K. Kwong , S. M. Richardson , and C. H. Evans , “Chordin Knockdown Enhances the Osteogenic Differentiation of human Mesenchymal Stem Cells,” Arthritis Research & Therapy 10 (2008): R65, 10.1186/ar2436. 18533030 PMC2483456

[62] A. A. M. Rady , S. M. Hamdy , M. A. Abdel‐Hamid , M. G. A. Hegazy , S. A. Fathy , and A. A. Mostafa , “The Role of VEGF and BMP‐2 in Stimulation of Bone Healing with Using Hybrid Bio‐composite Scaffolds Coated Implants in Animal Model,” Bulletin of the National Research Centre 44 (2020): 131, 10.1186/s42269-020-00369-x.

[63] Y. Geng , H. Duan , L. Xu , et al., “BMP‐2 and VEGF‐A modRNAs in Collagen Scaffold Synergistically Drive Bone Repair through Osteogenic and Angiogenic Pathways,” Communications Biology 4 (2021): 82, 10.1038/s42003-020-01606-9. 33469143 PMC7815925

[64] C.‐J. Li , V. Madhu , G. Balian , A. S. Dighe , and Q. Cui , “Cross‐Talk between VEGF and BMP‐6 Pathways Accelerates Osteogenic Differentiation of Human Adipose‐Derived Stem Cells,” Journal of Cellular Physiology 230 (2015): 2671–2682, 10.1002/jcp.24983. 25753222

[65] Y. Huang , Y. Xu , S. Feng , P. He , B. Sheng , and J. Ni , “miR‐19b Enhances Osteogenic Differentiation of Mesenchymal Stem Cells and Promotes Fracture Healing through the WWP1/Smurf2‐mediated KLF5/β‐catenin Signaling Pathway,” Experimental & Molecular Medicine 53 (2021): 973–985, 10.1038/s12276-021-00631-w. 34035464 PMC8178348

[66] L. Shu , H. Zhang , B. F. Boyce , and L. Xing , “Ubiquitin E3 Ligase Wwp1 Negatively Regulates Osteoblast Function by Inhibiting Osteoblast Differentiation and Migration,” Journal of Bone and Mineral Research 28 (2013): 1925–1935, 10.1002/jbmr.1938. 23553732 PMC3749248

[67] S. Guan , Z. Zhang , and J. Wu , “Non‐coding RNA Delivery for Bone Tissue Engineering: Progress, Challenges, and Potential Solutions,” Iscience 25 (2022): 104807, 10.1016/j.isci.2022.104807. 35992068 PMC9385673

[68] X. Hu , S. Hipolito , R. Lynn , V. Abraham , S. Ramos , and F. Wong‐Staal , “Relative Gene‐silencing Efficiencies of Small Interfering RNAs Targeting Sense and Antisense Transcripts from the Same Genetic Locus,” Nucleic Acids Research 32 (2004): 4609–4617, 10.1093/nar/gkh790. 15333693 PMC516062

[69] S. W. Hong , Y. Jiang , S. Kim , C. J. Li , and D. Lee , “Target Gene Abundance Contributes to the Efficiency of siRNA‐mediated Gene Silencing,” Nucleic Acid Therapeutics 24 (2014): 192–198, 10.1089/nat.2013.0466. 24527979 PMC4026300

[70] A. Arvey , E. Larsson , C. Sander , C. S. Leslie , and D. S. Marks , “Target mRNA Abundance Dilutes microRNA and siRNA Activity,” Molecular Systems Biology 6 (2010): 363, 10.1038/msb.2010.24. 20404830 PMC2872614

[71] L. Chen , C. Bosmajian , and S. Woo , “Mechanistic Intracellular PK/PD Modeling to Inform Development Strategies for Small Interfering RNA Therapeutics,” Molecular Therapy Nucleic Acids 36 (2025): 102516, 10.1016/j.omtn.2025.102516. 40242045 PMC12002994

[72] I. V. Chernikov , U. A. Ponomareva , and E. L. Chernolovskaya , “Structural Modifications of siRNA Improve Its Performance in Vivo,” International Journal of Molecular Sciences 24 (2023): 956, 10.3390/ijms24020956. 36674473 PMC9862127

[73] Q. Li , M. Dong , and P. Chen , “Advances in Structural‐guided Modifications of siRNA,” Bioorganic & Medicinal Chemistry 110 (2024): 117825, 10.1016/j.bmc.2024.117825. 38954918

[74] A. A. Gorkun , D. P. Revokatova , I. M. Zurina , et al., “The Duo of Osteogenic and Angiogenic Differentiation in ADSC‐Derived Spheroids,” Frontiers in Cell and Developmental Biology 9 (2021): 572727, 10.3389/fcell.2021.572727. 33898413 PMC8063121

[75] J. Chen , L. Deng , C. Porter , et al., “Angiogenic and Osteogenic Synergy of Human Mesenchymal Stem Cells and Human Umbilical Vein Endothelial Cells Cocultured on a Nanomatrix,” Scientific Reports 8 (2018): 15749, 10.1038/s41598-018-34033-2. 30356078 PMC6200728

[76] D. N. Heo , M. Hospodiuk , and I. T. Ozbolat , “Synergistic Interplay between human MSCs and HUVECs in 3D Spheroids Laden in Collagen/Fibrin Hydrogels for Bone Tissue Engineering,” Acta Biomaterialia 95 (2019): 348–356, 10.1016/j.actbio.2019.02.046. 30831326

[77] M. C. Decarli , A. Seijas‐Gamardo , F. L. C. Morgan , et al., “Bioprinting of Stem Cell Spheroids Followed by Post‐Printing Chondrogenic Differentiation for Cartilage Tissue Engineering,” Advanced Healthcare Materials 12 (2023): 2203021, 10.1002/adhm.202203021. 37057819 PMC11468754

[78] A. C. Daly , M. D. Davidson , and J. A. Burdick , “3D bioprinting of High Cell‐density Heterogeneous Tissue Models through Spheroid Fusion within Self‐healing Hydrogels,” Nature Communications 12 (2021): 753, 10.1038/s41467-021-21029-2. PMC7854667 33531489

[79] I. Decoene , G. Nasello , R. F. Madeiro de Costa , et al., “Robotics‐Driven Manufacturing of Cartilaginous Microtissues for Skeletal Tissue Engineering Applications,” Stem Cells Translational Medicine 13 (2024): 278–292, 10.1093/stcltm/szad091. 38217535 PMC10940839

[80] D. Kang , S. Hong , S.‐J. Kim , H. Choi , K. Kim , and J. Jang , “Robotics‐assisted Modular Assembly of Bioactive Soft Materials for Enhanced Organ Fabrication,” Virtual and Physical Prototyping 19 (2024): 2390484, 10.1080/17452759.2024.2390484.

[81] A. M. Almeida , J. Mazeda , A. R. Pinho , M. C. Gomes , and J. F. Mano , “The Future of Automated Tissue Engineering: Robotic‐Assisted Strategies for Complex 3D Tissue Bottom‐Up Assembly,” Adv Materials Technologies 10 (2025): 70000, 10.1002/admt.202500210.

[82] S. Fuchs , A. Motta , C. Migliaresi , and C. J. Kirkpatrick , “Outgrowth Endothelial Cells Isolated and Expanded from human Peripheral Blood Progenitor Cells as a Potential Source of Autologous Cells for Endothelialization of Silk Fibroin Biomaterials,” Biomaterials 27 (2006): 5399–5408, 10.1016/j.biomaterials.2006.06.015. 16837042

[83] M. I. Santos , S. Fuchs , M. E. Gomes , R. E. Unger , R. L. Reis , and C. J. Kirkpatrick , “Response of Micro‐ and Macrovascular Endothelial Cells to Starch‐based fiber Meshes for Bone Tissue Engineering,” Biomaterials 28 (2007): 240–248, 10.1016/j.biomaterials.2006.08.006. 16945411

[84] R. Sanz‐Horta , A. Matesanz , A. Gallardo , et al., “Technological Advances in Fibrin for Tissue Engineering,” Journal of Tissue Engineering 14 (2023): 20417314231190288, 10.1177/20417314231190288. 37588339 PMC10426312

[85] S. Wein , C. Schemmer , M. A. Al Enezy‐Ulbrich , et al., “Fibrin‐Based Hydrogels with Reactive Amphiphilic Copolymers for Mechanical Adjustments Allow for Capillary Formation in 2D and 3D Environments,” Gels 10 (2024): 182, 10.3390/gels10030182. 38534600 PMC10970602

[86] F. Lee and M. Kurisawa , “Formation and Stability of Interpenetrating Polymer Network Hydrogels Consisting of Fibrin and Hyaluronic Acid for Tissue Engineering,” Acta Biomaterialia 9 (2013): 5143–5152, 10.1016/j.actbio.2012.08.036. 22943886

[87] P. Alamán‐Díez , E. García‐Gareta , M. Arruebo , and M. Á. Pérez , “A Bone‐on‐a‐chip Collagen Hydrogel‐based Model Using Pre‐differentiated Adipose‐derived Stem Cells for Personalized Bone Tissue Engineering,” Journal of Biomedical Materials Research Part A 111 (2023): 88–105, 10.1002/jbm.a.37448. 36205241 PMC9828068

[88] S. Maji , M. Aliabouzar , C. Quesada , et al., “Ultrasound‐generated Bubbles Enhance Osteogenic Differentiation of Mesenchymal Stromal Cells in Composite Collagen Hydrogels,” Bioact Mater 43 (2025): 82–97, 10.1016/j.bioactmat.2024.09.018. 39345992 PMC11439547

