# Supplementary material for: siRNA Delivery via Cross‐Linked Gelatin Microparticles Enables Targeted Modulation of Osteogenic‐Vascular Cross‐Talk: An Advanced Human 3D in Vitro Test System for Therapeutic siRNA
Source: Adv Healthc Mater. 2026 Jan 22;15(13):e04773. doi: 10.1002/adhm.202504773 (PMC13058789; doi:10.1002/adhm.202504773)
Supplement: Supplementary file 1 — Supporting File: dhm70789‐sup‐0001‐SuppMat.pdf. [file ADHM-15-0-s001.pdf]

Supplementary Information

S1 Impact of cross-linking degree

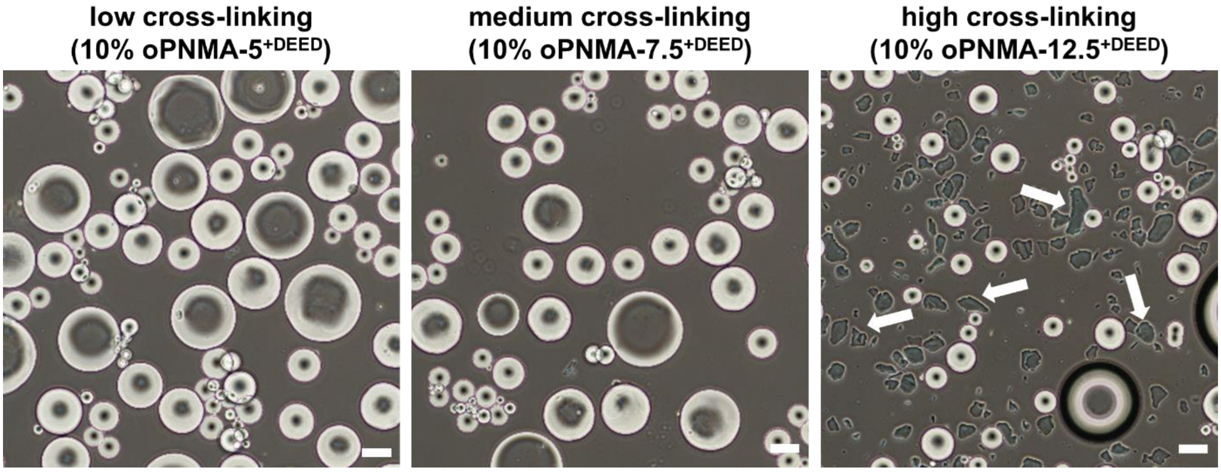

Figure S1. Microscopical analysis of cGM of different cross-linking degrees after loading with 4 µL concentrated siRNA-loaded CaP-NP. At highest cross-linking degree, crystal-like structures were observed indicated destabilization of CaP-NP at high cross-linking degree (indicated by white arrows). Scale bar: 50 µm

S2 Chordin silencing efficiency of cGM with medium cross-linking degree

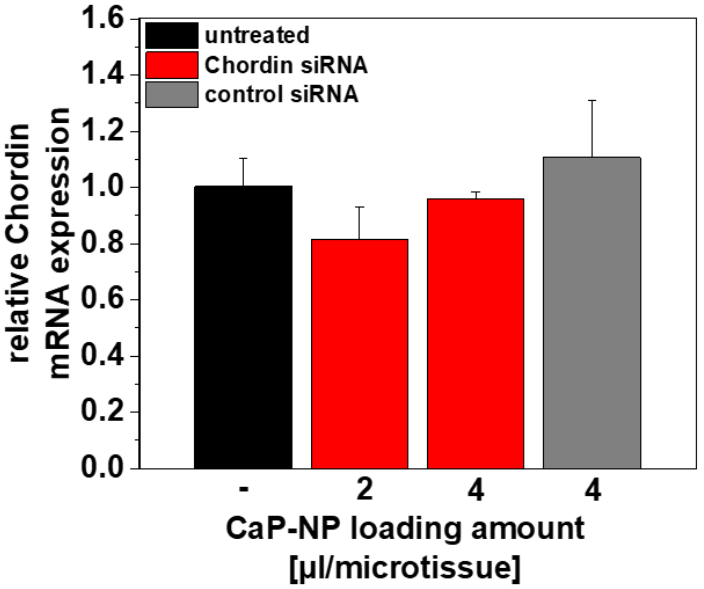

Figure S2. Analysis of siRNA-mediated Chordin silencing efficiency in osteogenic microtissues upon loading of cGM with a medium cross-linking degree (10% oPNMA-7.5^{+DEED}) with siRNA-carrying CaP-NP. Experimental set up: 1,875 L of siRNA-loaded CaP-NP were prepared and concentrated via ultrafiltration to a volume of 50 µL. Afterwards, 1.6 mg cGM (0.064 mg cGM/microtissue) were loaded with 50 (2 µl CaP-NP/microtissue) or 2x50 µL (4 µL CaP-NP/microtissue) of concentrated CaP-NP. 10^4 hMSCs were then aggregated with 0.064 mg cGM and osteogenic differentiation was induced by addition of osteogenic supplements. Chordin silencing efficiency was quantified via gene expression levels using one-step quantitative real-time PCR at day 4 of osteogenic differentiation. Data are presented as mean ± SD (n = 4). Statistically significant differences are indicated with (*) between the different groups ($p < 0.05$), two-way ANOVA with Tukey post hoc test. *CaP-NP:* oligomer-stabilized calcium phosphate nanoparticles; *mRNA:* messenger RNA; *siRNA:* small interfering RNA

S3 Impact of cGM amount on osteogenic differentiation

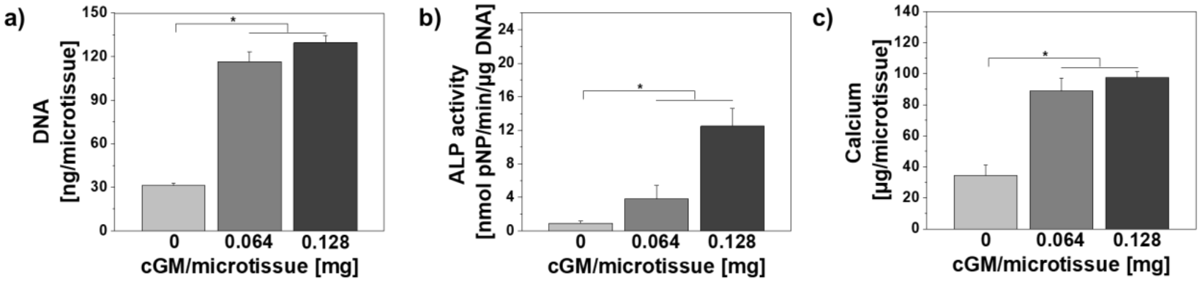

Figure S3. Effect of varying 10% oPNMA-5^{+DEED} cGM amounts/microtissue on proliferation and osteogenic differentiation of microtissues. Experimental setup: 10^4 hMSCs were aggregated with increasing amounts of cGM and cultured under osteogenic conditions for 14 days. **a)** DNA content of microtissues, assessed at day 7 as a measure of cell proliferation, increased significantly with higher cGM concentrations. **b)** ALP activity, measured at day 7 as an early osteogenic marker, was significantly enhanced by increasing cGM levels. **c)** Calcium content of microtissues, analyzed at day 14 as a measure of mineralization, was lowest in the absence of cGM, while incorporation of cGM led to significantly higher mineral deposition. Data are presented as mean ± SD (n = 4). Statistically significant differences are indicated with (*) between the different groups (p < 0.05), two-way ANOVA with Tukey post hoc test. *ALP:* alkaline phosphatase; *cGM:* cross-linked gelatine microparticles; *DNA:* deoxyribonucleic acid

S4 2D transfection of Chordin siRNA in hMSCs and effects on osteogenic differentiation

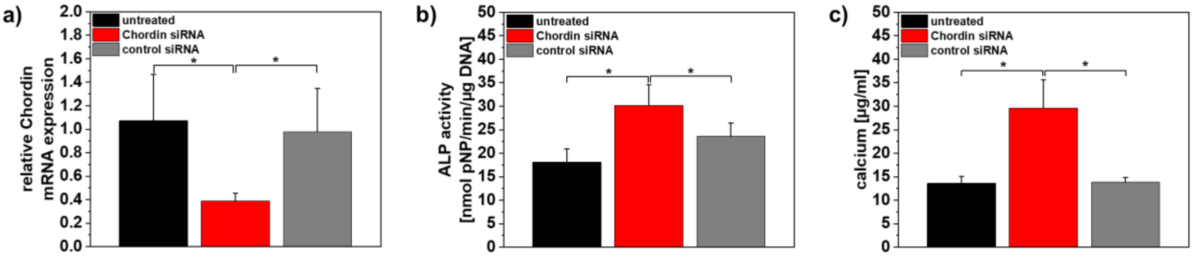

Figure S3 Analysis of siRNA-mediated Chordin silencing in 2D culture. Experimental set up: One day before transfection, hMSCs were seeded with a density of 5000 cells cm^{-2} into 48-well plates. At the next day, CaP-NP were prepared and 75 µL of CaP-NP solution was added per 48-well. Osteogenic differentiation was induced by addition of osteogenic supplements. **a)** Chordin silencing efficiency was quantified via gene expression levels using one-step quantitative real-time PCR at day 4 of osteogenic differentiation and shows successful Chordin silencing in hMSCs. **b)** Alkaline Phosphatase activity as an early osteogenic marker was quantified at day 4 of osteogenic differentiation. Analysis showed increased ALP activity in response to decreased Chordin expression. **c)** Calcium content as a late osteogenic differentiation marker was quantified at day 11 of osteogenic differentiation. We found that siRNA-mediated Chordin silencing significantly increased mineralization of hMSCs. Data are presented as mean ± SD (n = 4). Statistically significant differences are indicated with (*) between the different groups (p < 0.05), one-way ANOVA with Tukey post hoc test. *ALP:* alkaline phosphatase; *DNA:* deoxyribonucleic acid; *mRNA:* messenger RNA; *pNP:* para-nitrophenyl; *siRNA:* small interfering RNA
